# Prognostic value of glycaemic variability for mortality in critically ill atrial fibrillation patients and mortality prediction model using machine learning

**DOI:** 10.1186/s12933-024-02521-7

**Published:** 2024-11-26

**Authors:** Yang Chen, Zhengkun Yang, Yang Liu, Ying Gue, Ziyi Zhong, Tao Chen, Feifan Wang, Garry McDowell, Bi Huang, Gregory Y. H. Lip

**Affiliations:** 1 Liverpool Centre for Cardiovascular Science at University of Liverpool, Liverpool John Moores University and Liverpool Heart and Chest Hospital, Liverpool, UK; 2 Department of Cardiovascular and Metabolic Medicine, Institute of Life Course and Medical Sciences, University of Liverpool, Liverpool, UK; 3 Department of Cardiology, Tianjin Medical University General Hospital, Heping District, Tianjin, People’s Republic of China; 4 Department of Cardiovascular Medicine, The Second Affiliated Hospital, Jiangxi Medical College, Nanchang University, Nanchang, Jiangxi People’s Republic of China; 5 Department of Musculoskeletal Ageing and Science, Institute of Life Course and Medical Sciences, University of Liverpool, Liverpool, UK; 6 Department of Radiology, The First Affiliated Hospital, Zhejiang University School of Medicine, Hangzhou, Zhejiang People’s Republic of China; 7 Department of Computer Science, Aalto University, Espoo, Finland; 8 School of Pharmacy and Biomolecular Sciences, Liverpool John Moores University, Liverpool, UK; 9 Department of Cardiology, The First Affiliated Hospital of Chongqing Medical University, Chongqing, People’s Republic of China; 10 Department of Clinical Medicine, Danish Centre for Health Services Research, Aalborg University, 9220 Aalborg, Denmark; 11 Medical University of Bialystok, Bialystok, Poland

**Keywords:** Glycaemic variability, Atrial fibrillation, Intensive care unit, Mortality, Machine learning

## Abstract

**Background:**

The burden of atrial fibrillation (AF) in the intensive care unit (ICU) remains heavy. Glycaemic control is important in the AF management. Glycaemic variability (GV), an emerging marker of glycaemic control, is associated with unfavourable prognosis, and abnormal GV is prevalent in ICUs. However, the impact of GV on the prognosis of AF patients in the ICU remains uncertain. This study aimed to evaluate the relationship between GV and all-cause mortality after ICU admission at short-, medium-, and long-term intervals in AF patients.

**Methods:**

Data was obtained from the Medical Information Mart for Intensive Care IV 3.0 database, with admissions (2008–2019) as primary analysis cohort and admissions (2020–2022) as external validation cohort. Multivariate Cox proportional hazards models, and restricted cubic spline analyses were used to assess the associations between GV and mortality outcomes. Subsequently, GV and other clinical features were used to construct machine learning (ML) prediction models for 30-day all-cause mortality after ICU admission.

**Results:**

The primary analysis cohort included 8989 AF patients (age 76.5 [67.7–84.3] years; 57.8% male), while the external validation cohort included 837 AF patients (age 72.9 [65.3–80.2] years; 67.4% male). Multivariate Cox proportional hazards models revealed that higher GV quartiles were associated with higher risk of 30-day (Q3: HR 1.19, 95%CI 1.04–1.37; Q4: HR 1.33, 95%CI 1.16–1.52), 90-day (Q3: HR 1.25, 95%CI 1.11–1.40; Q4: HR 1.34, 95%CI 1.29–1.50), and 360-day (Q3: HR 1.21, 95%CI 1.09–1.33; Q4: HR 1.33, 95%CI 1.20–1.47) all-cause mortality, compared with lowest GV quartile. Moreover, our data suggests that GV needs to be contained within 20.0%. Among all ML models, light gradient boosting machine had the best performance (internal validation: AUC [0.780], G-mean [0.551], F1-score [0.533]; external validation: AUC [0.788], G-mean [0.578], F1-score [0.568]).

**Conclusion:**

GV is a significant predictor of ICU short-term, mid-term, and long-term all-cause mortality in patients with AF (the potential risk stratification threshold is 20.0%). ML models incorporating GV demonstrated high efficiency in predicting short-term mortality and GV was ranked anterior in importance. These findings underscore the potential of GV as a valuable biomarker in guiding clinical decisions and improving patient outcomes in this high-risk population.

**Supplementary Information:**

The online version contains supplementary material available at 10.1186/s12933-024-02521-7.

## Introduction

Atrial fibrillation (AF) is the most common persistent cardiac arrhythmia, affecting approximately one-third of individuals over time [1]. AF is associated with an increased risk of stroke, heart failure, various complications, hospitalization, and mortality, posing a substantial burden on healthcare systems [2, 3]. AF frequently complicates critical illness and is commonly seen in the Intensive Care Unit (ICU) [4]. Prospective and retrospective studies have reported the incidence of AF in ICU patients to range from 4.5–29.5% [4–8], and the 30-day all-cause mortality after ICU admission rate for AF patients was about 30% [9], highlighting the need to identify risk factors that contribute to this high mortality rate.

Both hyperglycemia and hypoglycemia are prevalent in hospitalized patients and are associated with increased complications and mortality in those with or without diabetes [10–12]. Recently, another metric of glucose control, known as glycaemic variability (GV), has been proposed to play a significant role in the disease-associated processes of dysglycemia [13]. GV refers to fluctuations in blood glucose levels around the mean and is considered a new marker of poor glycaemic control and increased complication risk [14]. In vitro and human studies have shown that GV induces more oxidative stress and endothelial dysfunction than sustained hyperglycemia [15, 16]. Notably, a large multicenter study found that GV is a stronger predictor of ICU mortality than mean glucose concentration [17]. Glycaemic control plays a vital role in managing AF [18], and recent studies indicate that stress hyperglycemia markers are associated with both short- and long-term mortality in AF patients [19]. However, the impact of GV on AF prognosis remains unclear.

Therefore, the primary objective of this study was to assess the relationship between GV and short-, intermediate-, and long-term all-cause mortality in patients with AF in the ICU. The exploratory aim was to evaluate the feasibility of GV in constructing an advanced machine learning (ML) model in predicting short-term all-cause mortality after ICU admission of AF patients.

## Methods

### Data source

This study utilized data from the Medical Information Mart for Intensive Care IV (MIMIC-IV, version 3.0), an extensive openly accessible database maintained by the computational physiology lab at the Massachusetts Institute of Technology [20]. The MIMIC-IV database is a significant and publicly available repository of de-identified electronic health records, documenting over 90,000 ICU admissions at the Beth Israel Deaconess Medical Center in Boston, Massachusetts, covering the period from 2008 to 2022. Since the Institutional Review Board (IRB) composed of the Massachusetts Institute of Technology and Beth Israel Deaconess Medical Center had approved the database for public use at its inception, additional informed consent for this study is not required (2001-P-001699/14; No.0403000206). One of the researcher (YC) had obtained permission to access this database (certificate number: 53753450), and has extensive experience related to the MIMIC database [21, 22]. All procedures involving human participants in this study were following the ethical standards of the institutional and national research committee and with the 1964 Helsinki declaration and its subsequent amendments or similar ethical standards.

### Study subjects

Patients diagnosed with AF on admission to the ICU were included. The International Classification of Diseases (ICD) version 9 code (427.31) and ICD version 10 codes (I48.X) were used for confirming AF. In this study, we chose temporal independent validation, which divides the dataset into a primary analysis cohort (in the ML section, it was further divided into training and internal validation cohort) and an external validation cohort according to the temporal order. This strategy is particularly suitable for checking the model’s ability to generalise on future data, as it simulates the actual performance of the model after deployment.

The primary analysis cohort consisted of AF patients admitted between 2008 and 2019, further divided into 70% training and 30% testing cohorts. Exclusion criteria for primary analysis cohort included: (i) age < 18 years; (ii) fewer than three blood glucose measurements; (iii) length of ICU stay < 24 h; (iv) multiple hospital or ICU admission records.

The external validation cohort comprised AF patients admitted between 2020 and 2022, to assess the generalizability of the model performance in a temporally distinct population. Exclusion criteria included: (i) diagnosed with severe acute respiratory syndrome coronavirus 2; (ii) age < 18 years; (iii) without records of ICU admission time; (iv) fewer than three blood glucose measurements during ICU stay; (v) length of ICU stay < 24 h; (vi) missing variables in the ML prediction model; (vii) multiple hospital or ICU admission records.

### Study outcomes

The primary outcomes included 30-day, 90-day, and 360-day all-cause mortality after ICU admission. In addition, the length of ICU stay and length of hospital stay were only for brief descriptive purposes. The main target outcome for machine learning (ML) model construction was 30-day all-cause mortality after ICU admission, given the critical nature of ICU care and the value of short-term mortality prediction in acute management and resource allocation.

### Covariates extraction

Collected covariates included demographic information (e.g. age, body mass index [BMI]), vital signs at 1st day after ICU admission (e.g. systolic blood pressure [SBP], arterial oxygen saturation [SpO2]), severity scores (sequential organ failure assessment [SOFA] and peripheral oxygen saturation [SAPS II]), comorbidities (e.g. chronic pulmonary disease, diabetes mellitus [DM]), laboratory results at 1st day after ICU admission (e.g. blood urea nitrogen [BUN], calcium), procedures and medications at 1st day after ICU admission (e.g. the use of vasopressor, the use of mechanical ventilation [MV]).

### Calculation of GV

In this analysis, blood glucose samples were collected exclusively during the ICU stay, ensuring that GV reflected the fluctuations in blood glucose levels specifically during the critical period of ICU. GV was assessed by calculating the coefficient of variation for blood glucose. The coefficient of variation is a measure of the variability of the data and is derived by dividing the standard deviation by the mean. Therefore, in this study, GV was calculated based on the formula GV = standard deviation of blood glucose / mean blood glucose × 100%.[23].

### Statistical analysis

The raw data extracted for this study had varying proportions of missing values (Supplementary Table S1), serum albumin was deleted due to missing values close to 70%. The remaining variable data with no more than 40% missing were processed by multiple interpolation via chained equations using the “miceforest” package in Python. This method leverages random forest imputation, which is effective in handling both numerical and categorical data while accounting for complex interactions among variables, and can effectively interpolate datasets with up to around 45% missing values [24]. By using this approach, we aimed to minimize potential biases and loss of statistical power due to data exclusion and ensure robust and reliable results. Then, we used the boxplot method to identify potential outliers, typically marking as outliers data points that exceeded 1.5 times the interquartile range (IQR). For our key exposure (GV measurement), we found that the points marked as outliers were concentrated at the higher end of the glucose measurement range, accounting for approximately 2% of all data points. Subsequently, to mitigate the effects of these extreme values, we applied a trimming (Winsorisation) technique to limit the glucose measurements to the 0th to 98th percentile.

The patients were categorized into four groups based on the 25th (13.2%), 50th (19.4%), and 75th (28.5%) percentiles of the GV distribution within our cohort as follows: Q1 (GV ≤ 13.2%); Q2 (13.2%< GV ≤ 19.4%; Q3 (19.4%< GV ≤ 28.5%); Q4 (GV > 28.5%). In this analysis, all continuous variables were non-normally distributed, and expressed using median and IQR, and differences among groups were compared using the Kruskal-Wallis test. In addition, categorical variables were expressed as counts with percentage (%), and analysed using Chi-square or Fisher’s exact tests.

To access the relationships between GV and outcomes, the restricted cubic splines (RCS) analysis was conducted to evaluate the relationships between GV and outcomes of interest, and to further explore whether there are non-linear relationships. When significant non-linear relationships were found, a two-piecewise Cox proportional hazards model was further performed to examine potential threshold effect (the hazard or protective effect is only apparent when the exposure has accumulated to a certain level) or saturation effect (when the exposure reaches a certain level, the hazard or protective effect does not continue to increase significantly) [25]. Moreover, we performed multivariate Cox proportional hazards models to calculate adjusted hazard ratios (HR) with 95% confidence intervals (CI). Further using three models for adjustments: Model 1 was unadjusted; Model 2 was adjusted for age, sex, race, BMI, vital signs (heart rate, respiratory rate, SBP, diastolic blood pressure [DBP], and SpO_2_), and severity scores (SOFA and SAPS II). Model 3 was Model 2 further adjusted for comorbidities (myocardial infarct, congestive heart failure, peripheral vascular disease, cerebrovascular accident, chronic pulmonary disease, renal disease, liver disease, DM, malignat cancer, and metastatic solid tumor), laboratory results (sodium, potassium, BUN, chloride, calcium, bicarbonate, estimated glomerular filtration rate [eGFR]), procedures (the use of renal replacement therapy, and MV), medications (vasopressor, angiotensin-converting enzyme inhibitor/ angiotensin II receptor blocker [ACEI/ARB], beta blocker, vitamin K antagonist, non-vitamin K antagonist oral anticoagulant, statin, and antiplatelet). We tested the proportional hazards assumption for the Cox proportional hazards models using Schoenfeld residuals. Then, we assessed the interaction between GV quartiles and each stratification factor (age [< 60 vs. ≥60 years], sex [males vs. females], BMI [< 30 vs. ≥30 kg/m^2^], and DM [DM vs. non-DM]) on mortality outcomes.

Furthermore, we applied several additional sensitivity analyses. First, to minimize the risk of reverse causality and ensure a more accurate assessment of the impact of GV on ICU mortality outcomes in AF patients, we excluded those died within the first three days after ICU admission and then regrouped according to the new quartiles of the GV. Second, to address the potential time-dependent effects of GV measurement, we performed a sensitivity analysis stratifying patients based on ICU length of stay (≤ 4 vs. >4 days), allowing us to assess the stability and predictive value of GV over different time frames. Third, we extended Model 3 by additionally adjusting for the total blood glucose measurement count and average sampling interval (calculated as the time difference between the first and last glucose measurements divided by the number of measurements minus one), to address potential biases arising from differences in sampling intensity across patients.

Additionally, to evaluate the prognostic value of GV relative to traditional glucose-related indicators (hyperglycaemia [defined as maximum blood glucose > 180 mg/dL during ICU stay] [26], and hypoglycaemia [defined as minimum blood glucose < 70 mg/dL during ICU stay]) [27], we compared their predictive performance for mortality outcomes. We assessed model performance using the area under the receiver operating characteristic curve with 95% CI and evaluated statistical differences using DeLong’s test.

Moreover, we used the “surv_cutpoint” function from the “survminer package” (version 0.4.9, R software) to determine optimal cut-off points of GV for different mortality outcomes based on the maximally selected rank statistics method, and to divide patients into two groups with significantly different mortality outcomes [28]. 

Previous machine learning methods have demonstrated superiority over traditional predictive scoring in the AF cohort [29]. In this section, we constructed a ML model to predict 30-day all-cause mortality after ICU admission for AF patients using a binary classification approach, to simplify the prediction task and focus on identifying high-risk patients within a fixed timeframe. We initially pre-evaluated the correlations between all variables using the Pearson correlation test, with a threshold of correlation coefficient (r) > 0.5 indicating moderate correlation. Variables with a correlation coefficient exceeding this threshold were excluded to mitigate potential multicollinearity issues, which could obscure the independent effects of key predictors, destabilize model parameter estimates, and affect the reliability of feature importance rankings. Following this, we split the whole cohort into a *training cohort* and an *internal validation cohort* in a ratio of 7:3. Then, three ML algorithms with default parameters were applied in the training cohort to rank the importance of features. For model construction, we selected the top 10 most important features identified by each of the three approaches, creating a combined set of features for following model construction based on their importance rankings. The selected features were again tested for Pearson’s correlation and additional variance inflation factor tests to avoid multicollinearity among features. Then, importing these features into seven common medical ML algorithms (e.g. light gradient boosting machine [LightGBM], random forest). We used random search with “RandomizedSearchCV” in Python for hyperparameters tuning of the ML models, which is a robust and efficient method, especially suitable for exploring a large hyperparameter space, as it randomly samples parameter combinations rather than testing them exhaustively. The optimal hyperparameters identified through this process are shown in Supplementary Table S2. Subsequently, in the *internal validation cohort* and *external validation cohort*, we plotted the ROC curves for ML models, SOFA and SAPS II, and comparing their performances based on various metrics (area under the curve [AUC], accuracy, specificity, F1-Score, G-mean, precision and recall). Last, for the highest performing ML model, we used SHapley Additive exPlanations (SHAP) to visualise the feature importance in the model and generated partial dependent plots to illustrate the relationship between the specific feature and outcome. Last, we built an easy-to-use online prediction platform according to the highest performing ML model to improve clinical accessibility.

All procedures of this analysis were conducted using SPSS software (version 26.0, USA), R (version 4.3.1, Austria), and Python (version 3.11.1, USA), with a *P-value* less than 0.05 considered statistically significant.

## Results

### Baseline characteristics of primary analysis cohort

A total of 8989 patients with AF were included (see Supplementary Fig. S1 for the flow chart), with a median (IQR) age of 76.5 (67.7, 84.3) years and 5193 (57.8%) males. The median (IQR) interval between each glucose measurement was 11.67 (8.71–14.75) hours, with a median (IQR) total blood glucose measurement count of 6 (4–11) times during the ICU stay. Additionally, the median (IQR) age of patients without three blood glucose tests during their ICU stay was approximately 74.96 (66.69–83.13) years, and 59.6% were male, but they had overall better clinical outcomes (e.g. 360-day all-cause mortality after ICU admission: 33.1%) (Supplementary Table S3).

**Fig. 1:**
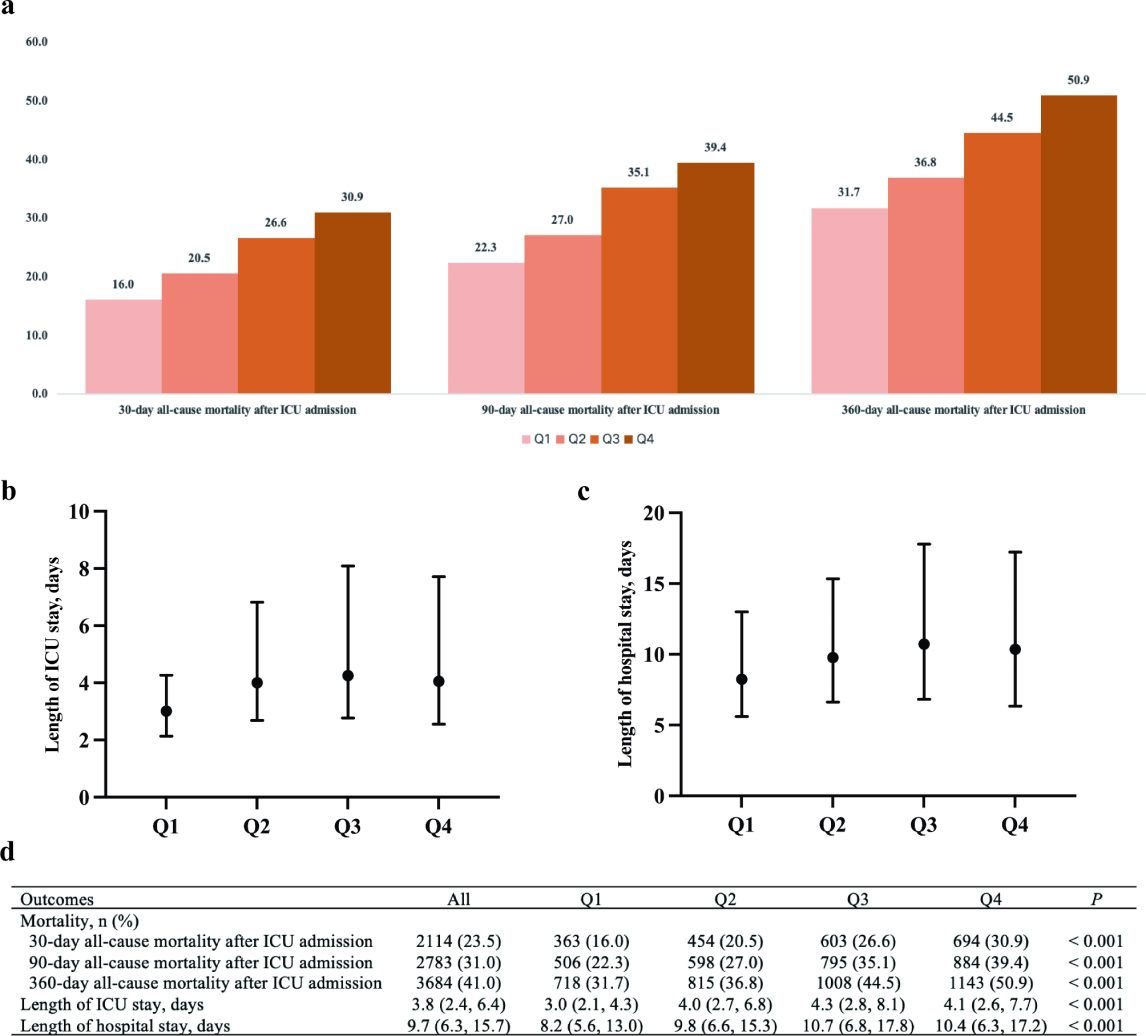
Outcomes of patients with atrial fibrillation according to quartiles of GV. Q1: GV ≤ 13.2%; Q2: 13.2% < GV ≤ 19.4%; Q3:19.4% < GV ≤ 28.5%; Q4: GV > 28.5%. ICU, intensive care unit; GV, glycaemic variability

According to Table 1, the higher level of GV group had higher heart rate, severity scores (SOFA and SAPS II), and more comorbidities (e.g. myocardial infarction, congestive heart failure, and DM). According to Fig. 1, all-cause mortality after ICU admission at 30, 90, and 360 days progressively increased across GV quartiles (Q1 to Q4, *P* < 0.001). Moreover, the higher levels of GV group had longer length of ICU or hospital stay (*P* < 0.001). Given that Q1 had the lowest mortality rates, it served as the reference group in the Cox proportional hazards models.

**Table 1:** Baseline characteristics of the quartiles of GV in critically ill patients with atrial fibrillation

Characteristic	All	Q1	Q2	Q3	Q4	*P*
N	8989	2266	2212	2265	2246	
Age, years	76.5 (67.7, 84.3)	76.8 (67.6, 84.7)	77.0 (68.1, 84.35)	76.0 (67.3, 84.0)	76.4 (67.8, 83.8)	0.125
Male, n (%)	5193 (57.8)	1336 (59.0)	1297 (58.6)	1283 (56.6)	1277 (56.9)	0.269
Race, n (%)						< 0.001
White	6567 (73.1)	1718 (75.8)	1644 (74.3)	1598 (70.6)	1607 (71.5)	
Asian	209 (2.3)	43 (1.9)	47 (2.1)	54 (2.4)	65 (2.9)	
Black	565 (6.3)	116 (5.1)	123 (5.6)	168 (7.4)	158 (7.0)	
Hispanic/Latino	177 (2.0)	32 (1.4)	39 (1.8)	46 (2.0)	60 (2.7)	
Other/unknown	1471 (16.4)	357 (15.8)	359 (16.2)	399 (17.6)	356 (15.9)	
Body mass index, kg/m^2^	28.7 (24.9, 33.4)	28.6 (24.9, 32.9)	28.6 (24.9, 33.3)	28.7 (25.0, 33.4)	28.9 (24.9, 33.8)	0.350
Vital sign						
Heart rate, beats/min	87.0 (75.0, 103.0)	85.0 (73.0, 100.0)	85.0 (75.0, 101.0)	87.0 (75.0, 103.0)	89.0 (76.0, 106.0)	< 0.001
Respiratory rate, beats/min	19.0 (15.0, 23.0)	18.0 (15.0, 22.0)	19.0 (15.0, 23.0)	19.0 (15.0, 23.0)	19.0 (16.0, 24.0)	< 0.001
SBP, mmHg	119.0 (104.0, 138.0)	121.0 (106.0, 140.0)	120.0 (105.0, 138.0)	118.0 (103.0, 136.0)	117.0 (102.0, 136.0)	< 0.001
DBP, mmHg	64.0 (54.0, 77.0)	66.0 (55.0, 78.0)	64.0 (54.0, 77.0)	63.0 (53.0, 76.0)	63.0 (53.0, 75.0)	< 0.001
SpO_2_, %	98.0 (95.0, 100.0)	98.0 (95.0, 100.0)	98.0 (95.0, 100.0)	98.0 (95.0, 100.0)	98.0 (95.0, 100.0)	0.285
Severity score						
SOFA	6.0 (3.0, 9.0)	4.0 (2.0, 7.0)	5.0 (3.0, 8.0)	6.0 (4.0, 10.0)	7.0 (4.0, 10.0)	< 0.001
SAPS II	41.0 (33.0, 50.0)	37.0 (31.0, 45.0)	40.0 (33.0, 48.0)	42.0 (35.0, 52.0)	45.0 (36.0, 54.0)	< 0.001
Comorbidity, n (%)						
Myocardial infarct	2175 (24.2)	450 (19.9)	478 (21.6)	602 (26.6)	645 (28.7)	< 0.001
Congestive heart failure	4648 (51.7)	1017 (44.9)	1095 (49.5)	1270 (56.1)	1266 (56.4)	< 0.001
Peripheral vascular disease	1498 (16.7)	333 (14.7)	345 (15.6)	393 (17.4)	427 (19.0)	< 0.001
Cerebrovascular disease	1648 (18.3)	467 (20.6)	435 (19.7)	392 (17.3)	354 (15.8)	< 0.001
Chronic pulmonary disease	2810 (31.3)	628 (27.7)	695 (31.4)	743 (32.8)	744 (33.1)	< 0.001
Renal disease	2810 (31.3)	548 (24.2)	643 (29.1)	747 (33.0)	872 (38.8)	< 0.001
Liver disease	947 (10.5)	152 (6.7)	206 (9.3)	282 (12.5)	307 (13.7)	< 0.001
Diabetes mellitus	3052 (34.0)	489 (21.6)	549 (24.8)	816 (36.0)	1198 (53.3)	< 0.001
Malignat cancer	1201 (13.4)	319 (14.1)	276 (12.5)	292 (12.9)	314 (14.0)	0.299
Metastatic solid tumor	506 (5.6)	144 (6.4)	124 (5.6)	129 (5.7)	109 (4.9)	0.186
Laboratory result at 1st day						
Sodium, mmol/L	139.0 (136.0, 141.0)	139.0 (136.0, 141.0)	139.0 (136.0, 141.0)	139.0 (136.0, 142.0)	138.0 (135.0, 141.0)	0.002
Potassium, mmol/L	4.2 (3.8, 4.7)	4.1 (3.8, 4.6)	4.2 (3.8, 4.6)	4.2 (3.8, 4.7)	4.3 (3.8, 4.8)	< 0.001
Blood urea nitrogen, mg/dL	24.0 (16.0, 40.0)	21.0 (15.0, 32.0)	23.0 (16.0, 35.0)	26.0 (17.0, 42.5)	28.5 (19.0, 48.0)	< 0.001
Chloride, mmol/L	104.0 (99.0, 108.0)	104.0 (100.0, 108.0)	104.0 (100.0, 108.0)	104.0 (99.0, 108.0)	103.0 (99.0, 108.0)	< 0.001
Calcium, mg/dL	8.3 (7.8, 8.8)	8.3 (7.9, 8.8)	8.3 (7.8, 8.8)	8.4 (7.9, 8.8)	8.3 (7.7, 8.8)	0.010
Bicarbonate, mmol/L	23.0 (20.0, 26.0)	24.0 (21.0, 26.0)	23.0 (21.0, 26.0)	23.0 (20.0, 26.0)	22.0 (19.0, 26.0)	< 0.001
eGFR, mL/min/1.73m^2^	56.5 (33.3, 82.5)	66.1 (43.1, 86.2)	60.7 (37.6, 84.3)	53.3 (30.7, 80.7)	46.3 (25.6, 74.6)	< 0.001
Procedure at 1st day, n (%)						
Renal replacement therapy	486 (5.4)	63 (2.8)	93 (4.2)	151 (6.7)	179 (8.0)	< 0.001
Mechanical ventilation	3889 (43.3)	742 (32.7)	966 (43.7)	1099 (48.5)	1082 (48.2)	< 0.001
Medication at 1st day, n (%)						
Vasopressor	4110 (45.7)	754 (33.3)	972 (43.9)	1180 (52.1)	1204 (53.6)	< 0.001
ACEI/ARB	457 (5.1)	131 (5.8)	103 (4.7)	103 (4.5)	120 (5.3)	0.188
Beta blocker	353 (3.9)	107 (4.7)	104 (4.7)	76 (3.4)	66 (2.9)	0.002
VKA	609 (6.8)	179 (7.9)	140 (6.3)	134 (5.9)	156 (6.9)	0.046
NOAC	248 (2.8)	70 (3.1)	66 (3.0)	62 (2.7)	50 (2.2)	0.294
Statin	2749 (30.6)	700 (30.9)	673 (30.4)	697 (30.8)	679 (30.2)	0.961
Antiplatelet agent	2980 (33.2)	677 (29.9)	725 (32.8)	796 (35.1)	782 (34.8)	< 0.001

Q1: GV ≤ 13.2%; Q2: 13.2% < GV ≤ 19.4%; Q3:19.4% < GV ≤ 28.5%; Q4: GV > 28.5%

ACEI, angiotensin-converting enzyme inhibitor; ARB, angiotensin II receptor blocker; DBP, diastolic blood pressure; eGFR, estimated glomerular filtration rate; GV, glycaemic variability; NOAC, non-vitamin K antagonist oral anticoagulant; SAPS II, simplified acute physiology score II; SBP, systolic blood pressure; SOFA, sequential organ failure assessment; SpO2, peripheral oxygen saturation; VKA, vitamin K antagonist

### Association of GV and 30-day all-cause motality after ICU admission

**Fig. 2:**
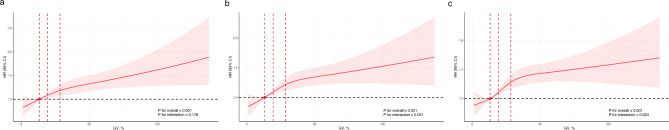
Restricted cubic spline analyses of GV and outcomes of interest. 30-day (**a**), 90-day (**b**), 360-day (**c**) all-cause mortality after ICU admission. The vertical red dashed lines from left to right represent the 25th (13.2%), 50th (19.4%), and 75th (28.5%) percentiles of the GV distribution. The shaded red area indicates the 95% confidence interval. GV, glycaemic variability

For 30-day all-cause mortality after ICU admission, the number of deaths and actual mortality rates observed in GV quartiles were as follows: Q1: 363 (16.0%); Q2: 454 (20.5%); Q3: 603 (26.6%); Q4: 694 (30.9%) (Fig. 1). Kaplan-Meier analysis (Supplementary Fig. S2a) revealed a significant decrease in 30-day survival across GV quartiles, with the order Q1 > Q2 > Q3 > Q4. As depicted in Fig. 2a, GV was linearly associated with an increased risk of 30-day all-cause mortality after ICU admission (*P-overall* < 0.001; *P non-linear* = 0.178). Based on the results of multivariate Cox proportional hazards models analysis in Table 2, higher quartiles (Q3 and Q4) of GV were significantly associated with higher risk of 30-day all-cause mortality after ICU admission (Q3: HR 1.19, 95% CI 1.04–1.37; Q4: HR 1.33, 95% CI 1.16–1.52). Subgroup analyses (Supplementary Table S4) revealed no significant interactions between GV and any of the subgroup factors.

**Table 2:** Cox proportional hazards models analyses for GV and mortality outcomes in critically ill patients with atrial fibrillation

Outcome		Model 1			Model 2			Model 3		
		HR (95%CI)	*P*	*P* for trend	HR (95%CI)	*P*	*P* for trend	HR (95%CI)	*P*	*P* for trend
30-day all-cause mortality after ICU admission										
	Q1	*Reference*		< 0.001	*Reference*		< 0.001	*Reference*		< 0.001
	Q2	1.31 (1.14, 1.50)	< 0.001		1.04 (0.90, 1.19)	0.609		1.08 (0.94, 1.24)	0.306	
	Q3	1.76 (1.54, 2.00)	< 0.001		1.18 (1.03, 1.35)	0.015		1.19 (1.04, 1.37)	0.011	
	Q4	2.13 (1.88, 2.42)	< 0.001		1.29 (1.13, 1.47)	< 0.001		1.33 (1.16, 1.52)	< 0.001	
90-day all-cause mortality after ICU admission										
	Q1	*Reference*		< 0.001	*Reference*		< 0.001	*Reference*		< 0.001
	Q2	1.25 (1.11, 1.40)	< 0.001		1.02 (0.91, 1.15)	0.728		1.06 (0.94, 1.19)	0.375	
	Q3	1.70 (1.52, 1.91)	< 0.001		1.23 (1.10, 1.38)	< 0.001		1.25 (1.11, 1.40)	< 0.001	
	Q4	2.01 (1.80, 2.24)	< 0.001		1.31 (1.17, 1.47)	< 0.001		1.34 (1.29, 1.50)	< 0.001	
360-day all-cause mortality after ICU admission										
	Q1	*Reference*		< 0.001	*Reference*		< 0.001	*Reference*		< 0.001
	Q2	1.21 (1.09, 1.34)	< 0.001		1.02 (0.93, 1.13)	0.643		1.05 (0.95, 1.16)	0.373	
	Q3	1.57 (1.42, 1.72)	< 0.001		1.21 (1.10, 1.34)	< 0.001		1.21 (1.09, 1.33)	< 0.001	
	Q4	1.90 (1.73, 2.09)	< 0.001		1.35 (1.22, 1.49)	< 0.001		1.33 (1.20, 1.47)	< 0.001	

Q1: GV ≤ 13.2%; Q2: 13.2% < GV ≤ 19.4%; Q3:19.4% < GV ≤ 28.5%; Q4: GV > 28.5%

Model 1 was unadjusted

Model 2 was adjusted for age, sex, race, body mass index, vital signs (heart rate, respiratory rate, systolic blood pressure, diastolic blood pressure, arterial oxygen saturation), and severity scores (sequential organ failure assessment, simplified acute physiology score II)

Model 3 was Model 2 further adjusted for comorbidities (myocardial infarct, congestive heart failure, peripheral vascular disease, cerebrovascular accident, chronic pulmonary disease, renal disease, liver disease, diabetes mellitus, malignat cancer, and metastatic solid tumor), laboratory results (sodium, potassium, blood urea nitrogen, chloride, calcium, bicarbonate, estimated glomerular filtration rate), procedures (the use of renal replacement therapy, and mechanical ventilation), medications (vasopressor, angiotensin-converting enzyme inhibitor/ angiotensin II receptor blocker, beta blocker, vitamin K antagonist, non-vitamin K antagonist oral anticoagulant, statin, and antiplatelet agent)

CI, confidence interval; GV, glycaemic variability; HR, hazard ratio; ICU, intensive care unit

### Association between GV and 90-day all-cause motality after ICU admission

In terms of 90-day all-cause mortality after ICU admission, the observed deaths and corresponding mortality rates for the GV quartiles were: Q1 with 506 deaths (22.3%), Q2 with 598 deaths (27.0%), Q3 with 795 deaths (35.1%), and Q4 with 884 deaths (39.4%) (Fig. 1). Kaplan-Meier curve (Supplementary Fig. 2b) shows that the cumulative 90-day survival rate of AF patients was significantly and progressively lower in the order of Q1 > Q2 > Q3 > Q4. Figure 2b suggests a non-linear relationship between GV and risk of 90-day all-cause mortality after ICU admission (*P-overall* < 0.001; *P non-linear* = 0.021), although the overall pattern was largely linear. Based on Table 2, higher quartiles (Q3 and Q4) of GV were significantly associated with higher risk of 90-day all-cause mortality after ICU admission (Q3: HR 1.25, 95% CI 1.11–1.40; Q4: HR 1.34, 95% CI 1.29–1.50). Subgroup analyses (Supplementary Table S5) showed no significant interaction between GV and each of subgroup factors.

### Association of GV and 360-day all-cause motality after ICU admission

The distribution of 360-day all-cause mortality across GV quartiles was as follows: Q1 reported 718 deaths (31.7%), Q2 had 815 deaths (36.8%), Q3 recorded 1,008 deaths (44.5%), and Q4 showed 1,143 deaths (50.9%) (Fig. 1). Kaplan-Meier curve (Supplementary Fig. 2c) shows that the cumulative 360-day survival rate of AF patients was significantly and progressively lower in the order of Q1 > Q2 > Q3 > Q4. Figure 2c suggests a non-linear relationship between GV and 360-day all-cause mortality after ICU admission (*P-overall* < 0.001; *P non-linear* = 0.004), though the general trend remained close to linear. Table 2 suggests that higher quartiles (Q3 and Q4) of GV are significantly associated with higher risk of 360-day all-cause mortality after ICU admission (Q3: HR 1.21, 95% CI 1.09–1.33; Q4: HR 1.33, 95% CI 1.20–1.47). Subgroup analyses (Supplementary Table S6) showed significant interaction between GV and BMI < 30 kg/m^2^ or BMI ≥ 30 kg/m^2^ (*P for interaction* = 0.011), suggesting a stronger relationship between GV and long-term mortality in patients with obesity.

### Sensitivity analysis

Supplementary Table S7 shows that the associations between GV and mortality outcomes among patients with AF patients remained stable and similar after excluding patients who died within three days of ICU admission.

Supplementary Table S8 presents that the relationships between GV and mortality outcomes varied by ICU length of stay (≤ 4 days vs. >4 days). In patients with longer ICU stays, the associations between higher quartiles of GV (Q3 and Q4) and increased mortality outcomes were more pronounced, with significant interaction effects (30-day: *P for interaction* < 0.001; 90-day: *P for interaction* = 0.016; 90-day: *P for interaction* = 0.002).

After further adjustment for total blood glucose measurement count and average sampling interval, the associations between higher quartiles (Q3 and Q4) of GV and mortality outcomes showed consistent results with the primary analysis, supporting the stability of our findings (Supplementary Table S9).

### Comparison of GV with hyperglycemia and hypoglycemia

Supplementary Table S10 demonstrates that GV outperformed both hyperglycemia and hypoglycemia in predicting 30-day and 90-day all-cause mortality after ICU admission, with statistically significant differences (30-day: GV vs. hyperglycaemia [*P* < 0.001], GV vs. hyporglycaemia [*P* < 0.001]; 90-day: GV vs. hyperglycaemia [*P* = 0.003], GV vs. hyporglycaemia [*P* < 0.001]). For 360-day all-cause mortality, although GV showed a higher area under the receiver operating characteristic curve compared to both hyperglycemia and hypoglycemia, the difference was not statistically significant when compared with hyperglycemia (*P* = 0.091) but remained significant when compared with hypoglycemia (*P* < 0.001).

### Optimal risk stratification cut-off points for GV on mortality outcomes

For 30-day, 90-day, and 360-day all-cause mortality after ICU admission outcomes, the optimal cut-off points of GV were 18.6%, 18.3%, and 18.3%, respectively (Supplementary Fig. S3). Considering clinical utility and data volatility, we set the optimal risk stratification cut-off point at 20.0%. Supplementary Table S11 shows that the risk of each mortality outcomes was significantly higher for GV > 20.0% compared with GV ≤ 20.0%.

### Saturation effect analysis of GV on mortality outcomes

As there were significant non-linear relationships between GV and all mortality outcomes except for 30-day all-cause mortality after ICU admission, and a clear inflection point was observed in each of Fig. 2b and c with increasing GV, with a relatively plateaued trend after the inflection point. Therefore, we performed saturation effect analysis. According to Supplementary Table S12, we found significant saturation effects. Specifically, the inflection points for 90-day and 360-day all-cause mortality after ICU admission as outcomes corresponding to GV were 49.6% and 48.7%, respectively, and the HR of each mortality outcome compared to the reference level increased significantly when the GV was below the inflection point, whereas the HR levelled off compared to the reference level when the GV exceeded the inflection point. In addition, actual deaths after ICU admission in the patients with a GV greater than 50% (*N* = 440) were higher than in the overall population at 30-day: 167 (38.0%), 90-day: 198 (45.0%), and 360-day: 243 (55.2%), respectively. Overall, these results suggest that GV around 50.0% is a critical cut-off point beyond which effect of further increases in glycaemic volatility on the mortality risk tends to saturate.

### Feature pre-selection for the ML models

The heatmap of all variables illustrating their correlations is provided in Supplementary Fig. S4. Due to serum chloride and sodium (*r* = 0.69), eGFR and renal disease (*r* = -0.58) or BUN (*r* = -0.67), SBP and DBP (*r* = 0.54), serum chloride, eGFR, and DBP were excluded in the following process. In the *training cohort*, three ML algorithms with default parameters pre-selected the 20 features used to construct the model (Supplementary Fig. S5), of which GV was in the top ten in each algorithm. Importantly, there was no strong correlation or multicollinearity among the selected features (Supplementary Fig. S6).

### ML models construction and evaluation

**Fig. 3:**
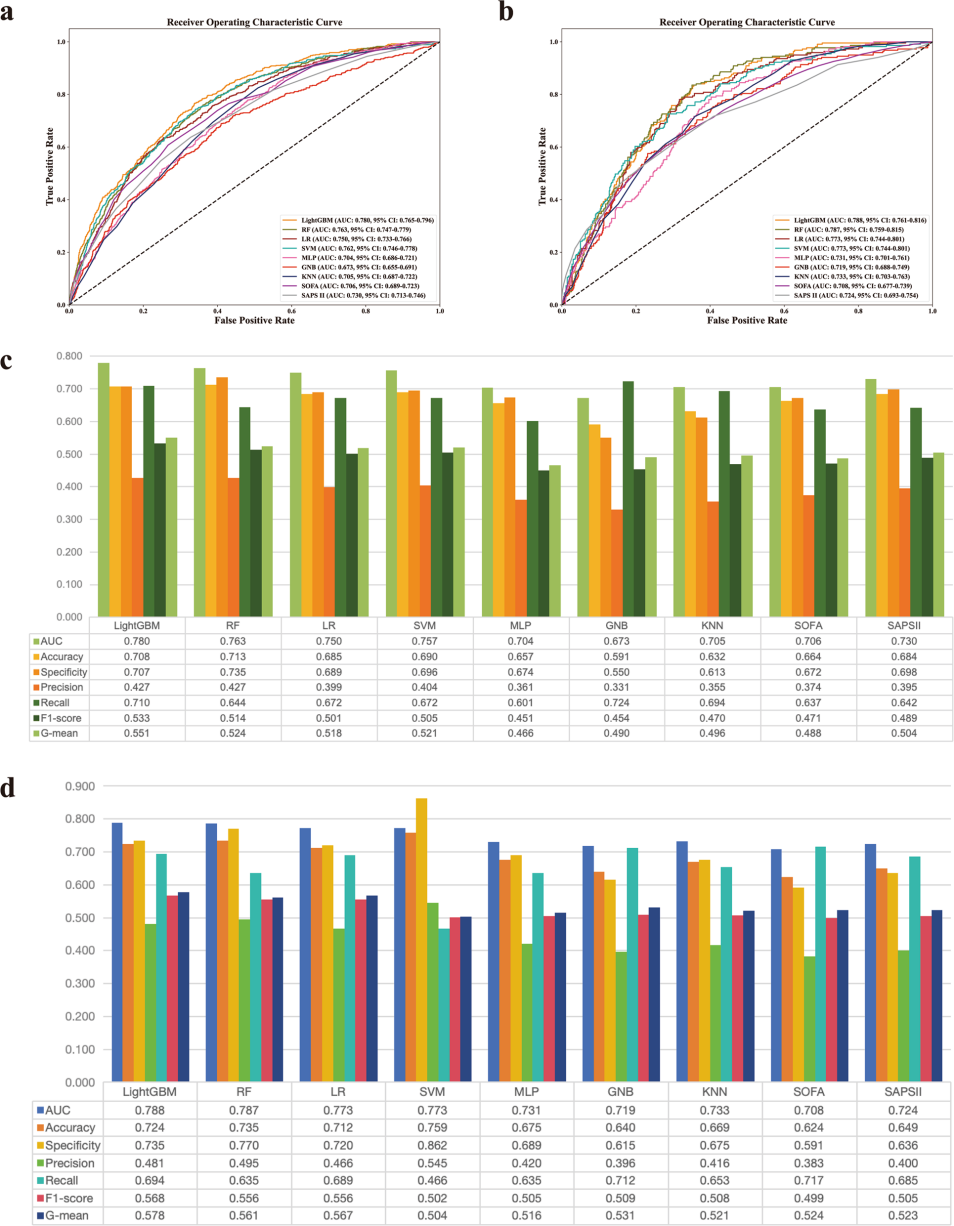
Evaluation the performance of machine learning models, SOFA and SAPS II. ROC curve (**a**) and other metrics (**c**) in the internal validation. ROC curve (**b**) and other metrics (**d**) in the external validation. AUC, area under curve; CI, confidence interval; GNB, gaussian naive bayes; KNN, k-nearest neighbors; LightGBM, light gradient boosting machine; LR, logistic regression; MLP, multilayer perceptron; RF, random forest; ROC, receiver operating characteristic; SAPS II, simplified acute physiology score II; SOFA, sequential organ failure assessment; SVM, support vector machine

After inputting the selected features into seven ML models, the optimal hyperparameters were determined. Then, the ROC curves (Fig. 3a), and other metrics (Fig. 3c) of all ML models were evaluated in the *internal validation cohort*. LightGBM was considered the best model because it had the highest AUC (0.780), F1-score (0.533), and G-mean (0.551), and outperformed the traditional scores SOFA and SAPS II.

In the external validation cohort, a total of 837 AF patients were included (median age 72.9 years [65.3–80.2], 67.4% male), other baseline characteristics are shown in Supplementary Table S13. Additionally, Fig. 3b and d show the ROC curves and other metrics for all ML models in the external validation. LightGBM remained best performing with the highest AUC (0.788), F1 score (0.568) and G-mean (0.578).

### Visualization of feature importance

**Fig. 4:**
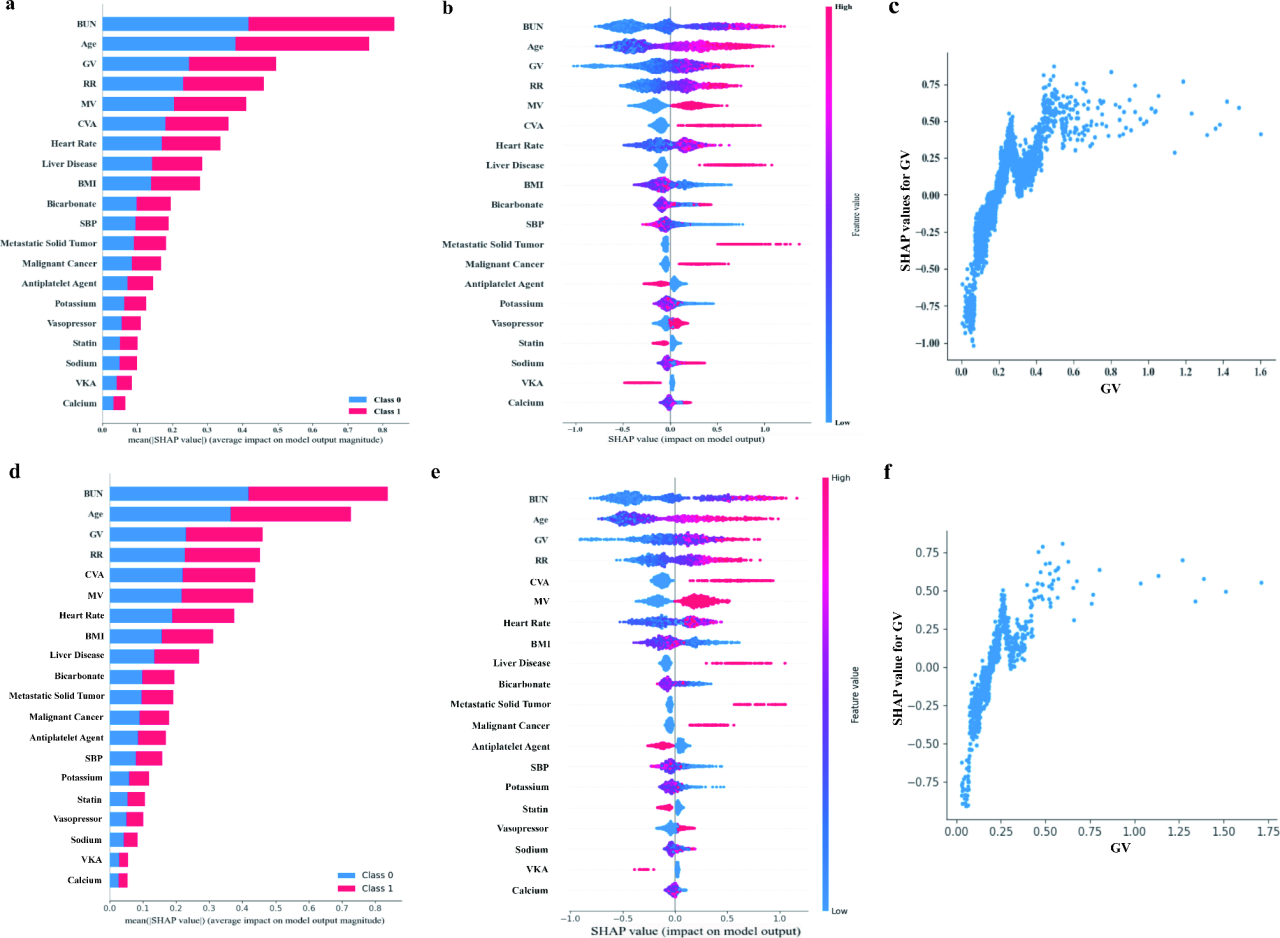
SHAP values of all features in the light gradient boosting machine model. In the internal validation, ordering of SHAP values for all features of histograms (**a**) and scatterplots (**b**), and dependence plot of GV (**c**). In the external validation, ordering of SHAP values for all features of histograms (**d**) and scatterplots (**e**), and dependence plot of GV (**f**). BMI, body mass index; BUN, blood urea nitrogen; CVA, cerebrovascular accident; GV, glycaemic variability; ICU, intensive care unit; MV, mechanical ventilation; RR, respiratory rate; SBP, systolic blood pressure; SHAP, SHapley Additive exPlanations; VKA, vitamin K antagonist

We calculated and ranked the corresponding SHAP values for each feature in the internal validation cohort for the LightGBM model (Fig. 4a and b), with GV being in the third significance compared to the other predictors. The bias dependence plot (Fig. 4c) showed that increased GV was linked to higher risk of 30-day all-cause mortality after ICU admission in AF patients. Moreover, the SHAP values for all features in the *external validation cohort* for the LightGBM model are shown in Fig. 4d and e, the GV is also ranked third. The bias dependence plot (Fig. 4f) also showed that increased GV was linked to higher risk of 30-day all-cause mortality after ICU admission in AF patients.

### Web-based prediction platform

To enhance the usability of our LightGBM model, we embedded it in a user-friendly web page that facilitates validation or prediction by external users and practitioners. The web site is “http://162.62.58.247:8008/”. As an example, Supplementary Fig. S7 illustrates the case of a 68-year AF patient with the displayed characteristics. The final output probability of 30-day all-cause mortality after ICU admission is 0.77, suggesting at high-risk.

## Discussion

We retrospectively assessed the effect of GV on short-term, medium-term, and long-term all-cause mortality in patients with critically ill AF in a large ICU database, and the main findings were as follows: (i) High levels of GV were significantly associated with higher risks of 30-day, 90-day, and 360-day all-cause mortality after ICU admission, with a trend toward a linear association. And our data suggests that a GV level of 20.0% may serve as an optimal cut-off point for mortality risk stratification, helping clinicians identify patients who could benefit from closer monitoring. (ii) The analysis revealed that the impact of GV on mortality was influenced by the length of ICU stay, significant interaction effects were observed across 30-day, 90-day, and 360-day mortality outcomes, indicating that the prognostic value of GV may be amplified in patients requiring prolonged intensive care. (iii) We generated a LightGBM model consisting of GV and other clinical parameters beyond the traditional critical illness score, with a high contribution of GV to LightGBM. Overall, our findings highlight that GV has an important role in the management of mortality risk stratification in critically ill AF patients and that attention needs to be paid to the more dramatic glycaemic fluctuations in AF patients.

From a physiological perspective, glucose metabolism plays a crucial role in cardiovascular function, as the heart primarily relies on glucose for energy [30]. The metabolic processing of glucose is thus essential for maintaining the cardiovascular system’s physiological integrity. Disruptions to this metabolic equilibrium, especially within diseased cardiac tissues, can serve as pivotal catalysts for the onset and progression of cardiovascular disorders. Several studies have consistently demonstrated a correlation between fasting blood glucose-related glycaemic changes and the occurrence or prognosis of cardiovascular diseases, including heart attack, stroke, and myocardial infarction [31–34]. This highlights the critical role of glycaemic instability in exacerbating the adverse prognosis of cardiovascular disease.

Previous research has demonstrated that large fluctuations in glucose levels promote the upregulation of markers associated with cardiac fibrosis, including collagen types I and III, and alpha-smooth muscle actin [35, 36]. These findings suggest that GV may contribute to AF development through oxidative stress, increased cardiomyocyte apoptosis, and atrial fibrosis. Additional studies propose that high GV could trigger AF by disrupting autonomic control of the heart or altering circulatory patterns [37, 38]. Emerging experimental evidence further indicates that large glucose oscillations can adversely affect atrial structure and electrical activity, providing a potential explanation for the link between GV and AF prognosis [39, 40]. 

DM or levels of blood glucose and the prognosis of AF patients have received extensive attention. Papazoglou et al. reported that DM was significantly associated with all-cause mortality (HR 1.40, 95% CI 1.11–1.75) and cardiovascular mortality (HR 1.39, 95% CI 1.07–1.81) in patients with AF during mean of 2.6 years follow-up [41]. However, In addition, Akirov and colleagues showed that the risk of all-cause mortality was significantly higher in the group with admission glucose 141-199 mg/dL (HR 2.10, 95% CI 1.19–7.94) and the group with admission glucose more than 200 mg/dL (HR 1.60, 95% CI 1.02–5.31) in patients with AF compared with the group with admission glucose 70-110 mg/dL, but significance disappeared in AF patients with DM [42]. These findings may underline the importance of focusing on dysglycaemia in the prognosis of AF patients. Moreover, Sim et al. suggested that blood glucose fluctuations measured as the difference between the highest and lowest levels of blood glucose were associated with the development of post-operative AF [43]. However, this measurement of glucose fluctuation may be limited in that the highest and lowest values focus only on the extremes of blood glucose fluctuations and ignore fluctuations in the middle values. This means that even if blood glucose values change dramatically between two measurements, this method does not reflect this. Such an approach may underestimate or overestimate the reality of GV. To our knowledge, no studies have examined the relationship between GV, as defined by fluctuations across all glucose measurements, and mortality in critically ill AF patients. Our findings suggest that GV outperforms both hyperglycemia and hypoglycemia in predicting mortality outcomes, underscoring the importance of monitoring glycemic fluctuations rather than relying solely on isolated glucose levels. This highlights the need for continuous glucose monitoring to detect harmful fluctuations in ICU AF patients.

Notably, the relationship between GV and risk of short-term mortality differed between DM and non-DM patients, despite no significant interaction being observed. This suggests that the impact of GV during the initial phase may be diminished by early interventions and acute management strategies commonly employed in DM patients, such as insulin treatment [44]. Thus, the prognostic value of GV for short-term mortality after ICU admission should be interpreted with caution. Furthermore, the association between GV and long-term mortality was more pronounced in obesity patients. This may be related to metabolic characteristics specific to obesity patients, such as insulin resistance, inflammatory status and exacerbation of metabolic disorders [45, 46]. Chronic inflammatory response and metabolic dysfunction in obesity may further amplify the adverse prognostic impact of GV. Chronic inflammatory response and metabolic dysfunction in obesity status may further amplify the adverse prognostic impact of GV. This suggests the need for personalised glycaemic management for obesity AF patients, with more refined strategies to stabilise glycaemic fluctuations to reduce long-term mortality risk.

SAPS II and SOFA, as traditional ICU scoring systems, are commonly used for risk assessment [47, 48], but they have limitations in AF patients. SAPS II was originally developed using a non-cardiac ICU cohort, which reduces its applicability in AF populations. Additionally, SAPS II and SOFA lack comorbidities commonly associated with poor prognosis in patients with AF, and are unable to capture the multimorbidity patterns of AF [49]. How does our LightGBM model of short-term all-cause mortality for critically ill patients with AF compare with previous ones dedicated to AF patients? For short-term mortality of AF patients in general wards, Chen et al. constructed a predictive model using a Cox regression that was able to achieve an AUC of 0.834 [50]. Bisson et al. constructed a predictive model for mortality at one year after diagnosis of AF, and the AUC of their constructed ML model was approximately 0.785 [51]. But the performance of the models cannot be compared because the target predicted outcomes of the previous two are different from ours. Moreover, the AF-mortality prediction model of Samaras et al. predicted a longer follow-up period for the mortality event [52]. However, none of these models considered GV or other glucose-related biomarkers other than DM.

Our findings highlight the importance of focusing on glycaemic fluctuations for mortality risk management in patients with AF, which has implications for clinical treatment. In terms of pharmacological treatment, novel sodium-glucose cotransporter protein 2 inhibitors not only reduce blood glucose levels but also blood glucose fluctuations [53], and their application in AF patients effectively reduced AF recurrence [54]. Moreover, sodium-glucose cotransporter protein 2 inhibitors were also effective in reducing all-cause mortality in patients with prior AF and DM (HR 0.22, 95% CI 0.16–0.28) [55]. Otherwise, blood glucose fluctuations can cause electrolyte balance disorders [56], and hypokalaemia, hypomagnesaemia and hyperphosphataemia have been shown to be associated with the prognosis of AF [57]. Therefore, intensive monitoring of blood glucose fluctuations may be able to help prevent electrolyte disorders and thus improve the prognosis of patients with AF. Furthermore, current novel glucose monitoring technologies, both invasive and non-invasive [58], show great potential for future application in the prognostic management of patients with AF.

Our results suggest that a GV level of 20.0% may serve as an important tcut-off point for mortality risk stratification in critically ill AF patients. After GV exceeds 50.0%, we observed that the hazard ratio compared to the reference level did not increase significantly, suggesting a possible plateau effect in the risk association. However, the actual mortality rates in patients with GV > 50.0% were higher than in the patients with GV ≤ 50.0%. These findings indicate a higher actual mortality risk, highlighting the severity of outcomes in this subgroup, despite the lack of further increase in relative risk. Ultra-high GV may still increase the risk of other complications (e.g., infection, shock) that require ongoing attention and management. Moreover, further research is needed to understand why mortality risk plateaus beyond this point, potentially involving physiological adaptation mechanisms or the effects of interventions. These insights could guide the development of targeted treatment strategies.

Importantly, while GV provides valuable information for risk stratification, its utility may vary depending on the patient’s length of stay. For patients with extended ICU admissions, longer monitoring periods seem to enhance the detection of clinically relevant glycemic variability. For shorter ICU stays, we should interpret GV cautiously, as limited measurement time may underestimate glycemic fluctuations and their impact on mortality outcomes. Overall, the length of ICU stay should be considered when using GV as a predictive tool, and future studies may further explore optimal monitoring durations to balance measurement precision and clinical applicability. Moreover, the frequency of glucose measurements is critical for accurate GV assessment. Using a minimum of three measurements, as in our study, may miss complete dynamic glucose fluctuations, potentially limiting the precision of GV assessment. Continuous glucose monitoring offers a more comprehensive approach, capturing real-time changes and providing better insight into glycemic variability [59]. Future studies should consider continuous glucose monitoring integration to improve the accuracy of GV assessment.

### Strengths and limitations

This study has several strengths. First, it is one of the few to assess the impact of GV on the prognosis of AF patients in an ICU setting. Additionally, this study is the first to incorporate GV into a ML model for AF-related prognostic prediction, addressing a critical gap in the literature on glycaemic fluctuations and AF prognosis management. The analysis is supported by a large cohort of nearly 10,000 patients with a follow-up period of up to one year, ensuring a robust sample size and sufficient follow-up to enhance the generalisability of the findings. Another highlight is the development of a web-based LightGBM model platform, providing clinicians with an accessible tool for real-time prognosis evaluation.

However, this study has some limitations. First, as a retrospective study, it is subject to inherent biases, and certain confounding factors that may influence mortality were not accounted for in the initial design, potentially limiting the generalizability of the findings and the strength of causal interpretations. Second, retrospective designs can only establish associations rather than causality. While we found a strong association between GV and all-cause mortality, this does not confirm GV as a direct cause of death. Third, although each patient in the study had more than three glucose measurements during ICU stay, it remains unclear whether this is sufficient to accurately capture glycaemic fluctuations. Fourth, our study may have potential selection bias arising from the exclusion of patients with fewer than three blood glucose measurements, who demonstrated a lower mortality risk and shorter hospital stays, suggesting a milder disease course. This exclusion may have resulted in a cohort that is more representative of critically ill and closely monitored patients, which could affect the external validity of our findings when applied to a broader or less severe AF population. Fifth, another limitation is the lack of control for factors such as nutritional support during ICU stays or the use of medications that could affect blood glucose levels, which may have impacted the results. Sixth, our study is the focus on ICU patients, which may limit the generalizability of our findings to non-ICU settings. Seventh, this study also utilized the MIMIC IV database, spanning multiple years during which medical practices and standards of care may have evolved, potentially affecting the applicability of the findings to current clinical settings. Finally, while the LightGBM model performed well in the *internal validation and external validation*, they both originated from the same database and differ only in chronology, and further validation in independent cohorts from other sources is required to confirm our LightGBM’s validity.

## Conclusion

Higher levels of GV were significantly associated with short-term, medium-term, and long-term all-cause mortality after ICU admission in critically ill AF patients. GV levels play critical role in mortality risk stratification of AF patients arriving at ICU. This suggests that monitoring GV levels of AF patients in ICU may possibly be an important step for properly monitoring patients and deciding on future treatment.

## Electronic supplementary material

Below is the link to the electronic supplementary material.

Supplementary Material 1

## Data Availability

No datasets were generated or analysed during the current study.
