# Supplementary material for: Prognostic value of glycaemic variability for mortality in critically ill atrial fibrillation patients and mortality prediction model using machine learning
Source: Cardiovasc Diabetol. 2024 Nov 26;23:426. doi: 10.1186/s12933-024-02521-7 (PMC11590403; doi:10.1186/s12933-024-02521-7)
Supplement: Supplementary file 1 — Supplementary Material 1 [file 12933_2024_2521_MOESM1_ESM.pdf]

Supplementary Table S1 Missing values of variables in this study.

**Variables**	**Percentage of missing, n (%)**
Height	3478 (38.7)
Weight	2346 (26.1)
Heart rate	18 (0.2)
Respiratory rate	22 (0.2)
Systolic blood pressure	46 (0.5)
Diastolic blood pressure	46 (0.5)
Pulse oxygen saturation	18 (0.2)
Albumin	6217 (69.2)
Sodium	20 (0.2)
Potassium	25 (0.3)
Blood urea nitrogen	21 (0.2)
Creatinie	16 (0.2)
Chloride	14 (0.2)
Calcium	736 (8.2)
Bicarbonate	16 (0.2)

Supplementary Table S2 Best hyperparameters of each machine learning model.

**Classifiers**	**Hyperparameters**	
Light gradient boosting machine	n_estimators	360
	max_depth	7
	learning_rate	0.02
	boosting_type	gbdt
	objective	binary
	num_leaves	31
	colsample_bytree	0.8569864417588267
	min_child_samples	195
	subsample	0.57635394267667271
	class_weight	'balanced'
Random Forest	n_estimators	224
	max_depth	8
	criterion	‘gini’
	min_samples_leaf	23
	random_state	48
	min_sample_split	2
	class_weight	‘balanced’
Logistic Regression	C	10
	penalty	l2
	solver	‘sag’
	max_iter	200
	class_weight	‘balanced’
Support Vector Machine	C	10
	kernel	‘linear’
	probability	True
	tol	0.0001
	gamma	‘auto’
	class_weight	‘balanced’
Multilayer Perceptron	solver	‘adam’
	activation	‘relu’
	hidden_layer_sizes	(10, 10,)
	alpha	0.001
	max_iter	300
	random_state	3
Gaussian Naive Bayes	var_smoothing	1e-09
K-nearest Neighbors	n_neighbors	25
	p	2
	metric	manhattan
	weights	uniform

Supplementary Table S3. The study outcomes of atrial fibrillation patients excluded in this study.

Outcomes (N = 8168*)	Atrial fibrillation patients excluded in this study
Mortality, n (%)	
30-day all-cause mortality after ICU admission	1134 (13.9%)
90-day all-cause mortality after ICU admission	1752 (21.4%)
360-day all-cause mortality after ICU admission	2701 (33.1%)
Length of ICU stay, days	1.86 (1.28-3.22)
Length of hospital stay, days	7.86 (5.06-13.75)

*Based on basic exclusion criteria including (*i*) records of multiple hospital or ICU admission (N = 1556); (*ii*) length of ICU stay <24 hours (N = 3068).

Supplementary Table S4. Subgroup analysis for association of glycaemic variability and 30-day all-cause mortality after ICU admission.

**Subgroups**	**Number**	**Groups**	**HR (95% CI)**	***P***	***P for interaction***
Age < 60years	974	Q1	*Reference*		
		Q2	1.14 (0.65, 1.99)	0.658	
		Q3	1.31 (0.76, 2.26)	0.337	
		Q4	1.85 (1.05, 3.25)	0.032	
					0.995
Age ≥ 60years	8,015	Q1	*Reference*		
		Q2	1.06 (0.92, 1.23)	0.423	
		Q3	1.17 (1.02, 1.35)	0.028	
		Q4	1.30 (1.3, 1.50)	< 0.001	
Male	5,193	Q1	*Reference*		
		Q2	1.14 (0.94, 1.37)	0.174	
		Q3	1.19 (0.99, 1.43)	0.064	
		Q4	1.33 (1.10, 1.60)	0.003	
					0.527
Female	3,796	Q1	*Reference*		
		Q2	0.99 (0.80, 1.22)	0.914	
		Q3	1.16 (0.95, 1.43)	0.149	
		Q4	1.30 (1.06, 1.60)	0.011	
BMI < 30kg/m^2	5,229	Q1	*Reference*		
		Q2	0.96 (0.81, 1.14)	0.646	
		Q3	1.13 (0.96, 1.33)	0.159	
		Q4	1.25 (1.06, 1.48)	0.008	
					0.139
BMI ≥ 30kg/m^2	5,937	Q1	*Reference*		
		Q2	1.40 (1.09, 1.80)	0.009	
		Q3	1.38 (1.08, 1.78)	0.011	
		Q4	1.54 (1.20, 1.98)	< 0.001	
Diabetes	3,052	Q1	*Reference*		
		Q2	0.97 (0.73, 1.30)	0.858	
		Q3	1.05 (0.81, 1.37)	0.713	
		Q4	1.16 (0.91, 1.49)	0.233	
					0.505
Non-diabetes	5,937	Q1	*Reference*		
		Q2	1.11 (0.94, 1.30)	0.221	
		Q3	1.23 (1.05, 1.45)	0.012	
		Q4	1.41 (1.19, 1.67)	< 0.001	

Note: Q1: GV ≤ 13.2%; Q2: 13.2% < GV ≤ 19.4%; Q3:19.4% < GV ≤ 28.5%; Q4: GV > 28.5%. Cox proportional hazards model was adjusted for age, sex, race, body mass index, vital signs (heart rate, respiratory rate, systolic blood pressure, diastolic blood pressure, and arterial oxygen saturation), and severity scores (sequential organ failure assessment, and peripheral oxygen

saturation), comorbidities (myocardial infarct, congestive heart failure, peripheral vascular disease, cerebrovascular accident, chronic pulmonary disease, renal disease, liver disease, diabetes mellitus, malignat cancer, and metastatic solid tumor), laboratory results (sodium, potassium, blood urea nitrogen, chloride, calcium, bicarbonate, estimated glomerular filtration rate), procedures (the use of renal replacement therapy, and mechanical ventilation), medications (vasopressor, angiotensin-converting enzyme inhibitor/ angiotensin II receptor blocker, beta blocker, vitamin K antagonist, non-vitamin K antagonist oral anticoagulant, statin, and antiplatelet agent).

Abbreviations: BMI, body mass index; CI, confidence interval; HR, hazard ratio; ICU, intensive care unit.

Supplementary Table S5. Subgroup analysis for association of glycaemic variability and 90-day all-cause mortality after ICU admission.

**Subgroups**	**Groups**	**HR (95% CI)**		***P for interaction***
Age < 60years	Q1	*Reference*		
	Q2	1.15 (0.70, 1.90)	0.581	
	Q3	1.45 (0.90, 2.35)	0.127	
	Q4	2.21 (1.35, 3.60)	0.002	
				0.531
Age ≥ 60years	Q1	*Reference*		
	Q2	1.05 (0.93, 1.19)	0.457	
	Q3	1.23 (1.09, 1.39)	< 0.001	
	Q4	1.30 (1.15, 1.46)	< 0.001	
Male	Q1	*Reference*		
	Q2	1.13 (0.96, 1.33)	0.137	
	Q3	1.27 (1.09, 1.49)	0.003	
	Q4	1.40 (1.19, 1.64)	< 0.001	
				0.566
Female	Q1	*Reference*		
	Q2	0.96 (0.80, 1.15)	0.679	
	Q3	1.20 (1.01, 1.42)	0.040	
	Q4	1.26 (1.06, 1.50)	0.009	
BMI < 30kg/m^2	Q1	*Reference*		
	Q2	0.96 (0.83, 1.11)	0.616	
	Q3	1.15 (1.00, 1.33)	0.048	
	Q4	1.26 (1.009, 1.45)	0.001	
				0.066
BMI ≥ 30kg/m^2	Q1	*Reference*		
	Q2	1.32 (1.06, 1.64)	0.014	
	Q3	1.50 (1.21, 1.86)	< 0.001	
	Q4	1.56 (1.26, 1.94)	< 0.001	
Diabetes	Q1	*Reference*		
	Q2	1.03 (0.80, 1.32)	0.824	
	Q3	1.18 (0.94, 1.49)	0.144	
	Q4	1.28 (1.03, 1.59)	0.026	
				0.947
Non-diabetes	Q1	*Reference*		
	Q2	1.07 (0.93, 1.22)	0.352	
	Q3	1.27 (1.10, 1.45)	< 0.001	
	Q4	1.37 (1.19, 1.58)	< 0.001	

Note: Q1: GV ≤ 13.2%; Q2: 13.2% < GV ≤ 19.4%; Q3:19.4% < GV ≤ 28.5%; Q4: GV > 28.5%. Cox proportional hazards model was adjusted for age, sex, race, body mass index, vital signs (heart rate, respiratory rate, systolic blood pressure, diastolic blood pressure, and arterial oxygen saturation), and severity scores (sequential organ failure assessment, and peripheral oxygen

saturation), comorbidities (myocardial infarct, congestive heart failure, peripheral vascular disease, cerebrovascular accident, chronic pulmonary disease, renal disease, liver disease, diabetes mellitus, malignat cancer, and metastatic solid tumor), laboratory results (sodium, potassium, blood urea nitrogen, chloride, calcium, bicarbonate, estimated glomerular filtration rate), procedures (the use of renal replacement therapy, and mechanical ventilation), medications (vasopressor, angiotensin-converting enzyme inhibitor/ angiotensin II receptor blocker, beta blocker, vitamin K antagonist, non-vitamin K antagonist oral anticoagulant, statin, and antiplatelet agent).

Abbreviations: BMI, body mass index; CI, confidence interval; HR, hazard ratio; ICU, intensive care unit.

Supplementary Table S6. Subgroup analysis for association of glycaemic variability and 360-day ICU mortality.

**Subgroups**	**Groups**	**HR (95% CI)**	***P***	***P for interaction***
Age < 60years	Q1	*Reference*		
	Q2	1.13 (0.76, 1.67)	0.554	
	Q3	1.29 (0.88, 1.88)	0.199	
	Q4	1.91 (1.29, 2.81)	0.001	
				0.769
Age ≥ 60years	Q1	*Reference*		
	Q2	1.04 (0.94, 1.16)	0.450	
	Q3	1.20 (1.08, 1.33)	< 0.001	
	Q4	1.30 (1.17, 1.44)	< 0.001	
Male	Q1	*Reference*		
	Q2	1.09 (0.96, 1.25)	0.195	
	Q3	1.20 (1.05, 1.38)	0.008	
	Q4	1.38 (1.21, 1.58)	< 0.001	
				0.640
Female	Q1	*Reference*		
	Q2	1.00 (0.86, 1.16)	0.965	
	Q3	1.22 (1.05, 1.41)	0.009	
	Q4	1.27 (1.09, 1.47)	0.002	
BMI < 30kg/m^2	Q1	*Reference*		
	Q2	0.95 (0.84, 1.08)	0.436	
	Q3	1.09 (0.96, 1.23)	0.176	
	Q4	1.24 (1.10, 1.40)	< 0.001	
				0.011
BMI ≥ 30kg/m^2	Q1	*Reference*		
	Q2	1.30 (1.08, 1.56)	0.005	
	Q3	1.52 (1.27, 1.82)	< 0.001	
	Q4	1.56 (1.31, 1.87)	< 0.001	
Diabetes	Q1	*Reference*		
	Q2	1.07 (0.87, 1.32)	0.527	
	Q3	1.27 (1.05, 1.54)	0.016	
	Q4	1.39 (1.16, 1.67)	< 0.001	
				0.963
Non-diabetes	Q1	*Reference*		
	Q2	1.05 (0.93, 1.18)	0.449	
	Q3	1.19 (1.05, 1.34)	0.005	
	Q4	1.31 (1.16, 1.49)	< 0.001	

Note: Q1: GV ≤ 13.2%; Q2: 13.2% < GV ≤ 19.4%; Q3:19.4% < GV ≤ 28.5%; Q4: GV > 28.5%. Cox proportional hazards model was adjusted for age, sex, race, body mass index, vital signs (heart rate, respiratory rate, systolic blood pressure, diastolic blood pressure, and arterial oxygen saturation), and severity scores (sequential organ failure assessment, and peripheral oxygen

saturation), comorbidities (myocardial infarct, congestive heart failure, peripheral vascular disease, cerebrovascular accident, chronic pulmonary disease, renal disease, liver disease, diabetes mellitus, malignat cancer, and metastatic solid tumor), laboratory results (sodium, potassium, blood urea nitrogen, chloride, calcium, bicarbonate, estimated glomerular filtration rate), procedures (the use of renal replacement therapy, and mechanical ventilation), medications (vasopressor, angiotensin-converting enzyme inhibitor/ angiotensin II receptor blocker, beta blocker, vitamin K antagonist, non-vitamin K antagonist oral anticoagulant, statin, and antiplatelet agent).

Abbreviations: BMI, body mass index; CI, confidence interval; HR, hazard ratio; ICU, intensive care unit.

Supplementary Table S7. Association of GV and mortality outcomes after excluding patients who died within three days of ICU admission.

**Outcomes**	**Group**	**HR (95%CI)**	***P***	***P for trend***
30-day ICU all-cause mortality				
	Q1	*Reference*		
	Q2	1.08 (0.91, 1.27)	0.387	0.001
	Q3	1.23 (1.05, 1.45)	0.011	
	Q4	1.28 (1.08, 1.51)	0.004	
90-day ICU all-cause mortality				
	Q1	*Reference*		
	Q2	1.12 (0.97, 1.30)	0.121	< 0.001
	Q3	1.38 (1.20, 1.59)	< 0.001	
	Q4	1.37 (1.18, 1.58)	< 0.001	
360-day ICU all-cause mortality				
	Q1	*Reference*		
	Q2	1.11 (0.98, 1.26)	0.110	< 0.001
	Q3	1.34 (1.18, 1.51)	< 0.001	
	Q4	1.34 (1.18, 1.52)	< 0.001	

Note: Q1: GV ≤ 14.4%; Q2: 14.4% < GV ≤ 20.4%; Q3:20.4% < GV ≤ 29.1%; Q4: GV > 29.1%.

Cox proportional hazards model was adjusted for age, sex, race, body mass index, vital signs (heart rate, respiratory rate, systolic blood pressure, diastolic blood pressure, and arterial oxygen saturation), and severity scores (sequential organ failure assessment, and peripheral oxygen saturation), comorbidities (myocardial infarct, congestive heart failure, peripheral vascular disease, cerebrovascular accident, chronic pulmonary disease, renal disease, liver disease, diabetes mellitus, malignat cancer, and metastatic solid tumor), laboratory results (sodium, potassium, blood urea nitrogen, chloride, calcium, bicarbonate, estimated glomerular filtration rate), procedures (the use of renal replacement therapy, and mechanical ventilation), medications (vasopressor, angiotensin-converting enzyme inhibitor/ angiotensin II receptor blocker, beta blocker, vitamin K antagonist, non-vitamin K antagonist oral anticoagulant, statin, and antiplatelet agent).

Abbreviations: CI, confidence interval; GV, glycaemic variability; HR, hazard ratio; ICU, intensive care unit.

Supplementary Table S8. Sensitivity analysis for the relationships between GV and mortality outcomes varied by ICU length of stay (≤4 days vs. >4 days).

Outcome		HR (95% CI)	*P*	*P for interaction*
30-day all-cause mortality after ICU admission				
ICU length of stay ≤4 days	Q1	*Reference*		
	Q2	0.97 (0.79, 1.19)	0.766	
	Q3	1.09 (0.90, 1.32)	0.397	
	Q4	1.30 (1.08, 1.57)	0.006	
				< 0.001
ICU length of stay >4 days	Q1	*Reference*		
	Q2	1.12 (0.91, 1.38)	0.291	
	Q3	1.28 (1.05, 1.57)	0.016	
	Q4	1.30 (1.05, 1.60)	0.015	
90-day all-cause mortality after ICU admission				
ICU length of stay ≤4 days	Q1	*Reference*		
	Q2	0.91 (0.77, 1.08)	0.285	
	Q3	1.04 (0.89, 1.23)	0.605	
	Q4	1.21 (1.03, 1.42)	0.020	
				0.016
ICU length of stay >4 days	Q1	*Reference*		
	Q2	1.19 (0.99, 1.43)	0.066	
	Q3	1.46 (1.22, 1.75)	< 0.001	
	Q4	1.47 (1.22, 1.77)	< 0.001	
360-day all-cause mortality after ICU admission				
ICU length of stay ≤4 days	Q1	*Reference*		
	Q2	0.95 (0.83, 1.10)	0.498	
	Q3	1.03 (0.89, 1.18)	0.726	
	Q4	1.31 (1.15, 1.50)	< 0.001	
				0.002
ICU length of stay >4 days	Q1	*Reference*		
	Q2	1.12 (0.95, 1.31)	0.172	
	Q3	1.36 (1.17, 1.59)	< 0.001	
	Q4	1.36 (1.16, 1.60)	< 0.001	

Note: Q1: GV ≤ 13.2%; Q2: 13.2% < GV ≤ 19.4%; Q3:19.4% < GV ≤ 28.5%; Q4: GV > 28.5%. Cox proportional hazards model was adjusted for age, sex, race, body mass index, vital signs (heart rate, respiratory rate, systolic blood pressure, diastolic blood pressure, arterial oxygen saturation), and severity scores (sequential organ failure assessment, simplified acute physiology score II), comorbidities (myocardial infarct, congestive heart failure, peripheral vascular disease, cerebrovascular accident, chronic pulmonary disease, renal disease, liver disease, diabetes mellitus, malignat cancer, and metastatic solid tumor), laboratory results (sodium, potassium, blood urea nitrogen, chloride, calcium, bicarbonate, estimated glomerular filtration rate), procedures (the use of renal replacement therapy, and mechanical ventilation), medications (vasopressor, angiotensin-converting enzyme inhibitor/ angiotensin II receptor blocker, beta blocker, vitamin K antagonist, non-vitamin K antagonist oral anticoagulant, statin, and antiplatelet agent), and frequency of blood glucose measurements.

Abbreviations: CI, confidence interval; GV, glycaemic variability; HR, hazard ratio; ICU, intensive care unit.

Supplementary Table S9. Sensitivity analysis for GV and mortality outcomes after adjustment by Model 3 with total blood glucose measurement count and average sampling interval.

**Outcome**		**HR (95% CI)**	***P***	***P for trend***
30-day all-cause mortality after ICU admission				
	Q1	*Reference*		
	Q2	1.09 (0.95, 1.25)	0.228	< 0.001
	Q3	1.21 (1.06, 1.39)	0.006	
	Q4	1.30 (1.13, 1.49)	< 0.001	
90-day all-cause mortality after ICU admission				
	Q1	*Reference*		
	Q2	1.04 (0.92, 1.17)	0.506	< 0.001
	Q3	1.21 (1.08, 1.36)	0.001	
	Q4	1.28 (1.14, 1.44)	< 0.001	
360-day all-cause mortality after ICU admission				
	Q1	*Reference*		
	Q2	1.03 (0.93, 1.14)	0.602	< 0.001
	Q3	1.16 (1.05, 1.29)	0.003	
	Q4	1.27 (1.15, 1.41)	< 0.001	

Note: Q1: GV ≤ 13.2%; Q2: 13.2% < GV ≤ 19.4%; Q3:19.4% < GV ≤ 28.5%; Q4: GV > 28.5%. Cox proportional hazards model was adjusted for age, sex, race, body mass index, vital signs (heart rate, respiratory rate, systolic blood pressure, diastolic blood pressure, arterial oxygen saturation), and severity scores (sequential organ failure assessment, simplified acute physiology score II), comorbidities (myocardial infarct, congestive heart failure, peripheral vascular disease, cerebrovascular accident, chronic pulmonary disease, renal disease, liver disease, diabetes mellitus, malignat cancer, and metastatic solid tumor), laboratory results (sodium, potassium, blood urea nitrogen, chloride, calcium, bicarbonate, estimated glomerular filtration rate), procedures (the use of renal replacement therapy, and mechanical ventilation), medications (vasopressor, angiotensin-converting enzyme inhibitor/ angiotensin II receptor blocker, beta blocker, vitamin K antagonist, non-vitamin K antagonist oral anticoagulant, statin, and antiplatelet agent), total blood glucose measurement count, and average sampling interval.

Abbreviations: CI, confidence interval; GV, glycaemic variability; HR, hazard ratio; ICU, intensive care unit.

Supplementary Table S10. Comparison of predictive performance between GV with hyperglycaemia, and hypoglycaemia for mortality outcomes.

	AUC (95% CI)	*P*
Comparison of capacity to predict mortality outcomes alone		
30-day all-cause mortality after ICU admission		
GV	0.598 (0.588, 0.608)	Reference
Hyperglycaemia	0.569 (0.559, 0.579)	< 0.001
Hypoglycaemia	0.516 (0.506, 0.526)	< 0.001
90-day all-cause mortality after ICU admission		
GV	0.595 (0.584, 0.605)	Reference
Hyperglycaemia	0.576 (0.565, 0.586)	0.003
Hypoglycaemia	0.531 (0.520, 0.541)	< 0.001
360-day all-cause mortality after ICU admission		
GV	0.592 (0.581, 0.602)	Reference
Hyperglycaemia	0.581 (0.571, 0.592)	0.091
Hypoglycaemia	0.550 (0.540, 0.560)	< 0.001

Abbreviations: AUC, area under curve; CI, confidence interval; GV, glycaemic variability; ICU, intensive care unit.

Supplementary Table S11. Association of GV divided by 20% and mortality outcomes.

**Outcomes**	**Group**	**HR (95%CI)**	***P***
30-day all-cause mortality after ICU admission			
	GV ≤20.0%	*Reference*	
	GV >20.0%	1.2 (1.09, 1.32)	< 0.001
90-day all-cause mortality after ICU admission			
	GV ≤20.0%	*Reference*	
	GV >20.0%	1.25 (1.15, 1.36)	< 0.001
360-day all-cause mortality after ICU admission			
	GV ≤20.0%	*Reference*	
	GV >20.0%	1.24 (1.15, 1.33)	< 0.001

Cox proportional hazards model was adjusted for age, sex, race, body mass index, vital signs (heart rate, respiratory rate, systolic blood pressure, diastolic blood pressure, and arterial oxygen saturation), and severity scores (sequential organ failure assessment, and peripheral oxygen saturation), comorbidities (myocardial infarct, congestive heart failure, peripheral vascular disease, cerebrovascular accident, chronic pulmonary disease, renal disease, liver disease, diabetes mellitus, malignat cancer, and metastatic solid tumor), laboratory results (sodium, potassium, blood urea nitrogen, chloride, calcium, bicarbonate, estimated glomerular filtration rate), procedures (the use of renal replacement therapy, and mechanical ventilation), medications (vasopressor, angiotensin-converting enzyme inhibitor/ angiotensin II receptor blocker, beta blocker, vitamin K antagonist, non-vitamin K antagonist oral anticoagulant, statin, and antiplatelet agent).

Abbreviations: CI, confidence interval; GV, glycaemic variability; HR, hazard ratio; ICU, intensive care unit.

Supplementary Table S12. Saturation effect analysis of GV on 90-day and 360-day all-cause mortality after ICU admission.

	**Adjusted HR (95% CI)**	***P***
**90-day all-cause mortality after ICU admission**		
Fitting model by the standard Cox proportional hazards model	1.86 (1.51, 2.30)	< 0.001
Fitting model by the two-piecewise Cox proportional hazards model		
Inflection point	49.6%	
GV < 49.6%	2.90 (2.03, 4.30)	< 0.001
GV > 49.6%	1.01 (0.62, 1.64)	0.984
P for Log-likelihood ratio		0.002
**360-day all-cause mortality after ICU admission**		
Fitting model by the standard Cox proportional hazards model	1.80 (1.50, 2.17)	< 0.001
Fitting model by the two-piecewise Cox proportional hazards model		
Inflection point	48.7%	
GV < 48.7%	2.84 (2.08, 3.88)	< 0.001
GV > 48.7%	0.94 (0.61, 1.45)	0.780
P for Log-likelihood ratio		< 0.001

Cox proportional hazards model was adjusted for age, sex, race, body mass index, vital signs (heart rate, respiratory rate, systolic blood pressure, diastolic blood pressure, and arterial oxygen saturation), and severity scores (sequential organ failure assessment, and peripheral oxygen saturation), comorbidities (myocardial infarct, congestive heart failure, peripheral vascular disease, cerebrovascular accident, chronic pulmonary disease, renal disease, liver disease, diabetes mellitus, malignat cancer, and metastatic solid tumor), laboratory results (sodium, potassium, blood urea nitrogen, chloride, calcium, bicarbonate, estimated glomerular filtration rate), procedures (the use of renal replacement therapy, and mechanical ventilation), medications (vasopressor, angiotensin-converting enzyme inhibitor/ angiotensin II receptor blocker, beta blocker, vitamin K antagonist, non-vitamin K antagonist oral anticoagulant, statin, and antiplatelet agent).

Abbreviations: CI, confidence interval; GV, glycaemic variability; HR, hazard ratio; ICU, intensive care unit.

Supplementary Table S13. Baseline characteristics of patients in the *external validation cohort*.

	All (N = 837)	30-day ICU survivors (N = 618)	30-day ICU non-survivors (N = 219)	*P*
Age, years	72.91 (65.31, 80.21)	71.98 (64.47, 79.51)	75.40 (67.01, 83.11)	< 0.001
Male, n (%)	564 (67.4)	433 (70.1)	131 (59.8)	0.007
Body mass index, kg/m^2	28.12 (24.61, 32.82)	28.20 (24.69, 32.66)	27.83 (24.53, 33.39)	0.805
Heart rate, beats/min	85.00 (75.00, 101.00)	82.00 (74.00, 98.00)	92.00 (77.00, 108.00)	< 0.001
Respiratory rate, beats/min	19.00 (16.00, 23.00)	18.00 (16.00, 22.00)	21.00 (17.00, 26.00)	< 0.001
Systolic blood pressure, mmHg	116.00 (102.00, 133.00)	117.00 (103.00, 133.00)	116.00 (100.00, 131.00)	0.266
GV, %	18.88 (13.53, 26.80)	17.88 (12.67, 25.91)	22.99 (16.10, 30.78)	< 0.001
Severity score				
SOFA	6.00 (4.00, 9.00)	6.00 (3.00, 8.00)	9.00 (6.00, 12.00)	< 0.001
SAPS II	43.00 (34.00, 53.00)	40.00 (33.00, 50.00)	52.00 (42.00, 65.00)	< 0.001
Comorbidities, n (%)				
Cerebrovascular accident	178 (21.3)	118 (19.1)	60 (27.4)	0.012
Liver disease	77 (9.2)	39 (6.3)	38 (17.4)	< 0.001
Malignat cancer	111 (13.3)	67 (10.8)	44 (20.1)	0.001
Metastatic solid tumor	52 (6.2)	27 (4.4)	25 (11.4)	< 0.001
Laboratory result at 1st day				
Sodium, mmol/L	138.00 (135.00, 141.00)	138.00 (136.00, 140.00)	138.00 (134.00, 142.00)	0.971
Potassium, mmol/L	4.20 (3.80, 4.70)	4.30 (3.90, 4.70)	4.20 (3.70, 4.70)	0.282
Blood urea nitrogen, mg/dL	21.00 (15.00, 36.00)	20.00 (14.00, 32.00)	31.00 (21.00, 54.00)	< 0.001
Calcium, mg/dL	8.30 (8.00, 8.80)	8.30 (8.00, 8.80)	8.40 (7.90, 8.80)	0.559
Bicarbonate, mmol/L	21.00 (19.00, 24.00)	22.00 (20.00, 24.00)	21.00 (18.00, 25.00)	0.033
Procedure at 1st day, n (%)				
Mechanical ventilation	551 (65.8)	403 (65.2)	148 (67.6)	0.562
Medication at 1st day, n (%)				
Vasopressor	193 (23.1)	135 (21.8)	58 (26.5)	0.163

VKA	13 (1.6)	8 (1.3)	5 (2.3)	0.342
Statin	326 (38.9)	272 (44.0)	54 (24.7)	< 0.001
Antiplatelet agent	346 (41.3)	303 (49.0)	43 (19.6)	< 0.001

Abbreviations: GV, glycaemic variability; SAPS II, simplified acute physiology score II; SOFA, sequential organ failure assessment; VKA, vitamin K antagonist.

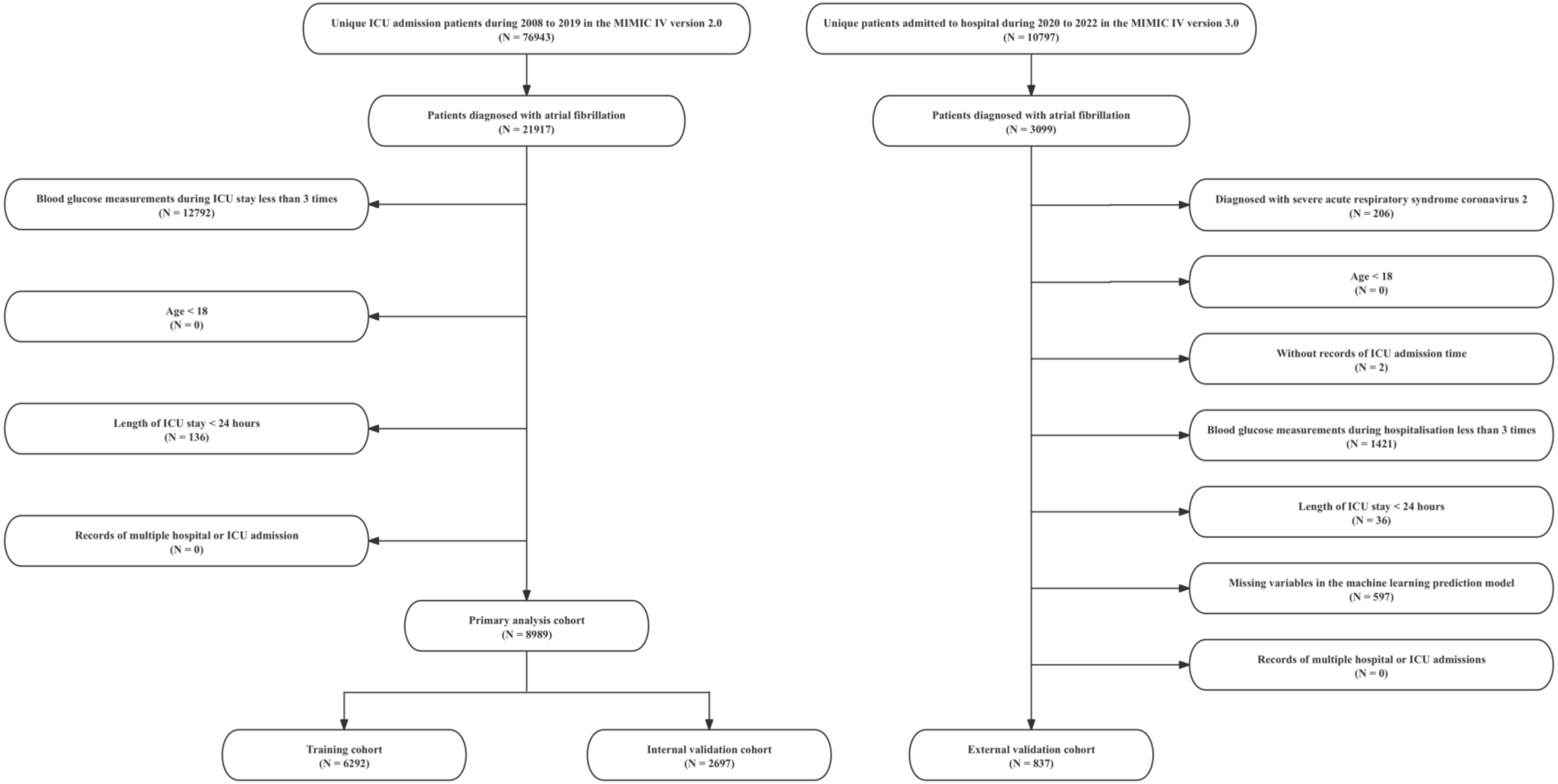

Supplementary Fig. S1 Flowchart of this study. ICU, intensive care unit; MIMIC IV, Medical Information Mart for Intensive Care IV.

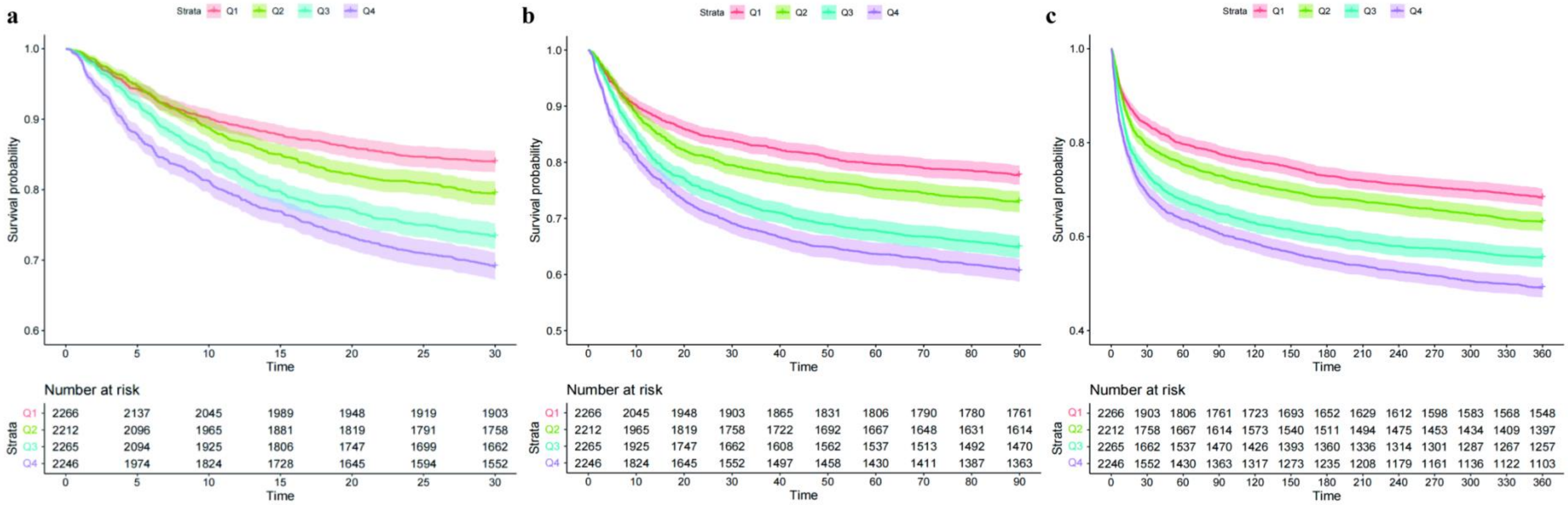

Supplementary Fig. S2 Kaplan-Meier curves of quartile GV groups for 30-day (a), 90-day (b), and 360-day (c) all-cause mortality after ICU admission. Q1: GV ≤ 13.2%; Q2: 13.2% < GV ≤ 19.4%; Q3:19.4% < GV ≤ 28.5%; Q4: GV > 28.5%. GV, glycaemic variability; ICU, intensive care unit.

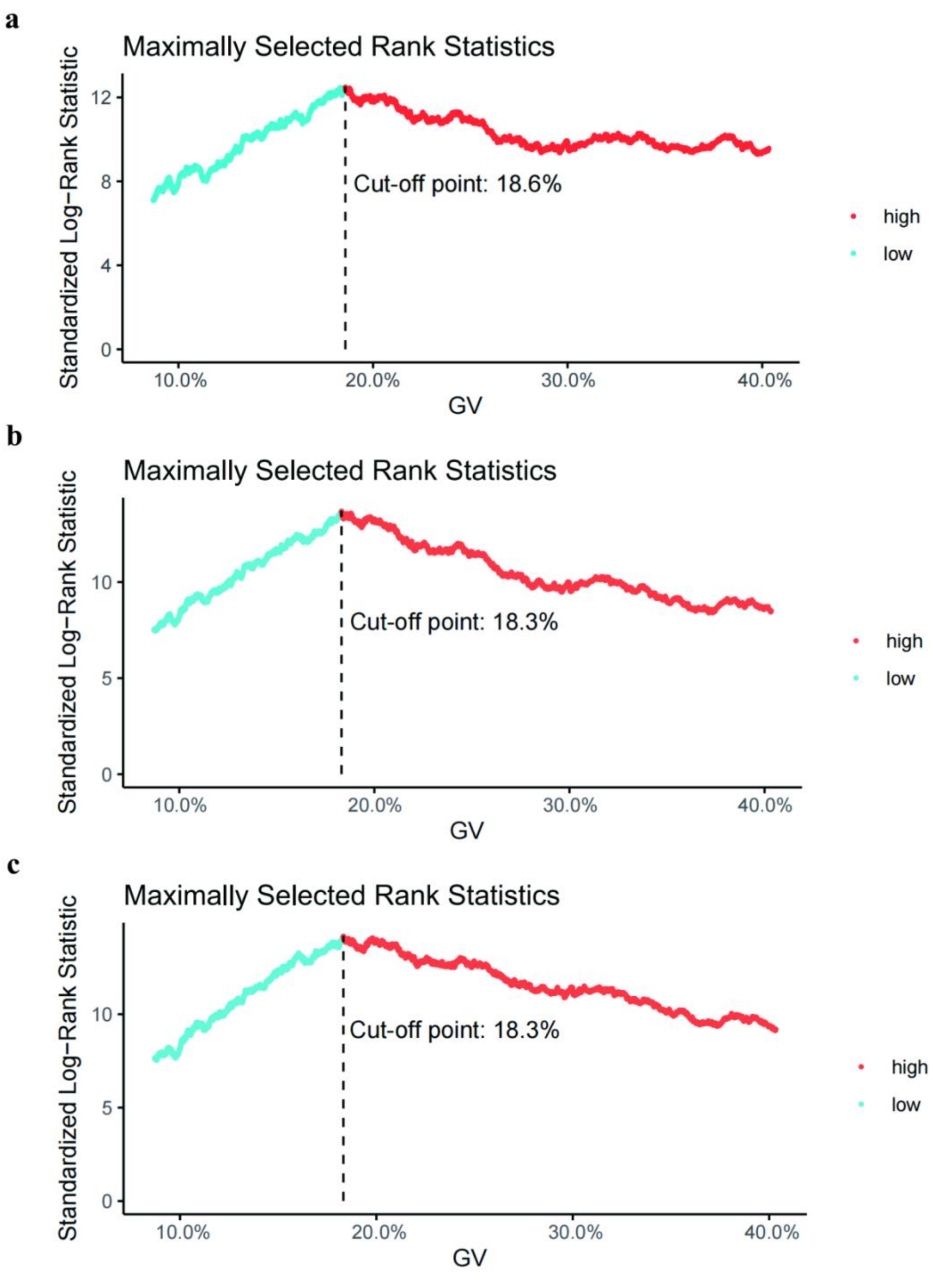

Supplementary Fig. S3 Cut-off points of glycaemic variability for 30-day (a), 90-day (b), and 360-day (c) all-cause mortality after intensive care unit admission.

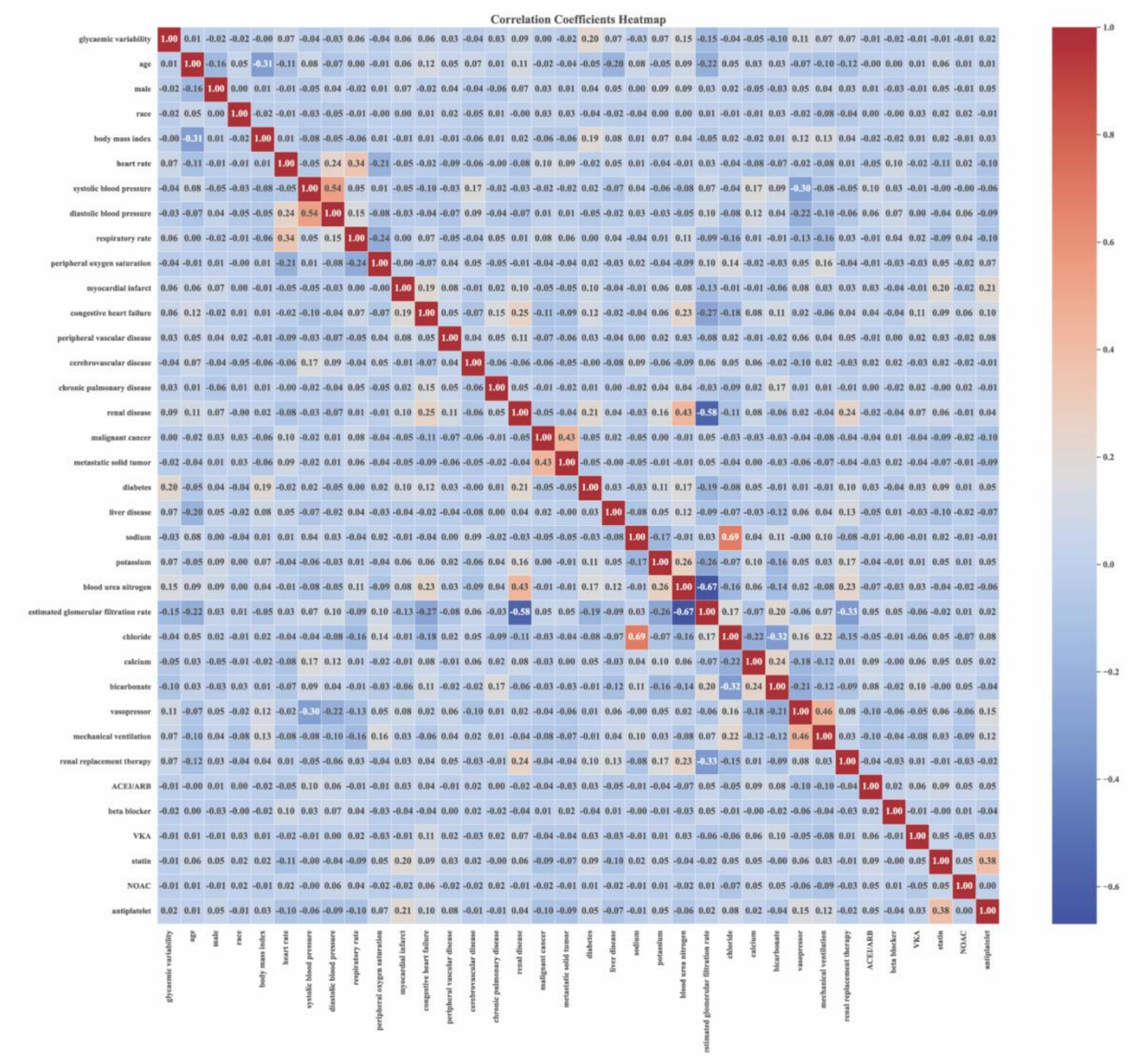

Supplementary Fig. S4 Correlation heatmap of all included variables. ACEI, angiotensin-converting enzyme inhibitor; ARB, angiotensin II receptor blocker; NOAC, non-vitamin K antagonist oral anticoagulant; VKA, vitamin K antagonist.

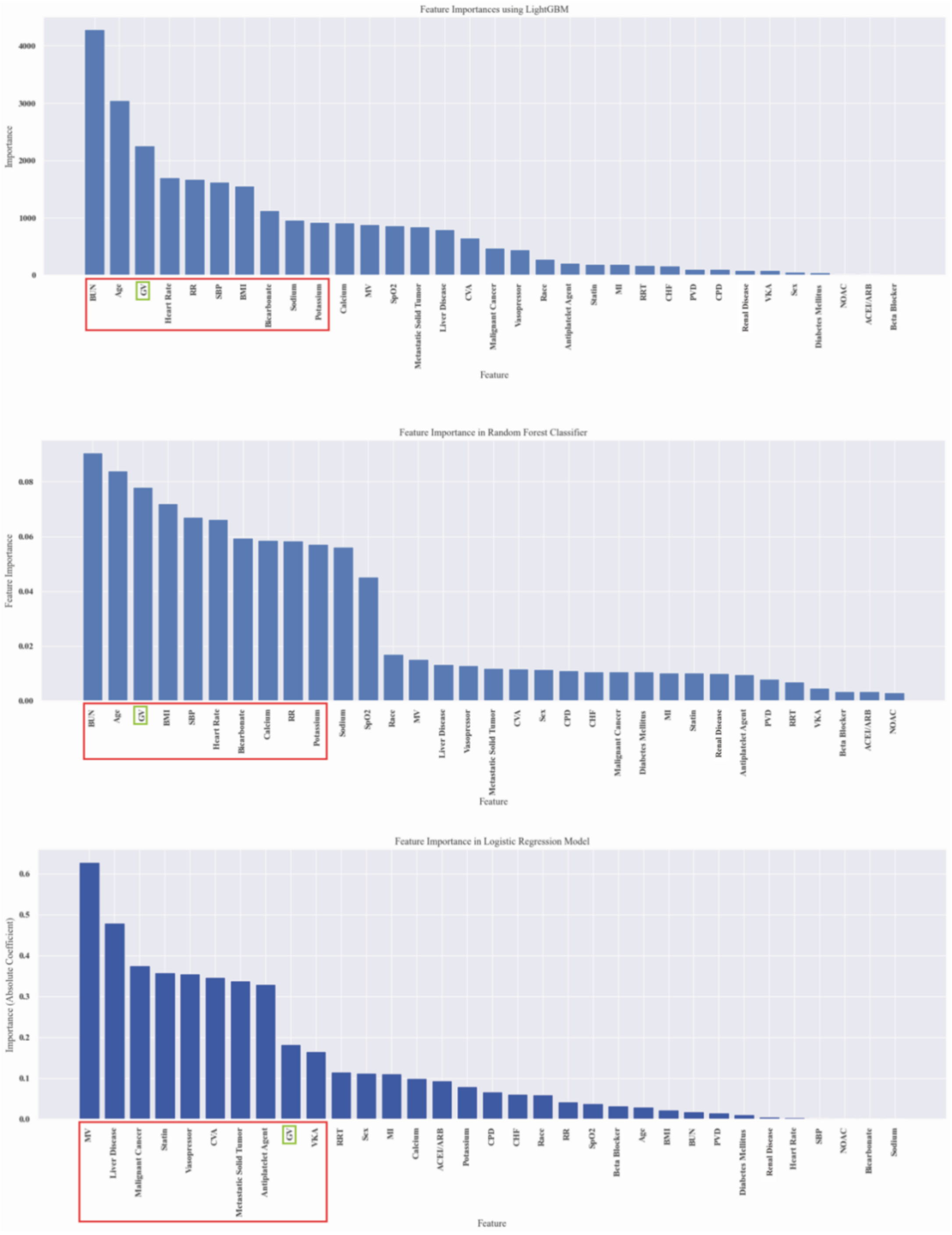

Supplementary Fig. S5 Feature selection for predicting 30-day all-cause mortality after ICU admission in patients with atrial fibrillation. The features in the red boxes are pre-selected by each machine learning algorithm, and the green boxes show the position in the importance ranking of the GV. ACEI, angiotensin-converting enzyme inhibitor; ARB, angiotensin receptor blocker; BMI, body mass index; BUN, blood urea nitrogen; CHF, congestive heart failure; CPD, chronic pulmonary disease; CVA, cerebrovascular accident; GV, glycaemic variability; ICU, intensive care unit; MI, myocardial infarction; MV, mechanical ventilation; NOAC, non-vitamin K antagonist oral anticoagulant; PVD, peripheral vascular disease; RR, respiratory rate; RRT, renal replacement therapy; SBP, systolic blood pressure; SpO_2, saturation of peripheral oxygen; VKA, vitamin K antagonist.

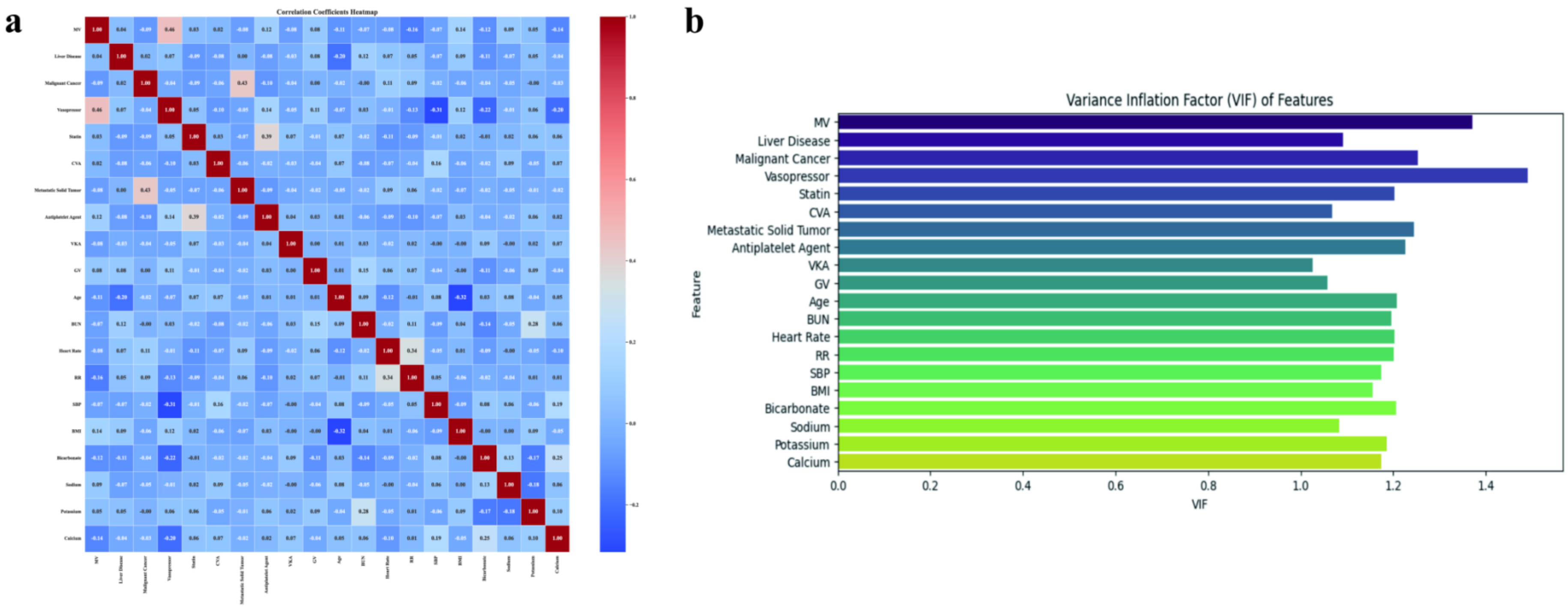

Supplementary Fig. S6 Pearson's correlation test (a) and variance inflation factor test (b) for selected features. BMI, body mass index; BUN, blood urea nitrogen; CVA, cerebrovascular accident; GV, glycaemic variability; MV, mechanical ventilation; RR, respiratory rate; SBP, systolic blood pressure; VKA, vitamin K antagonist.

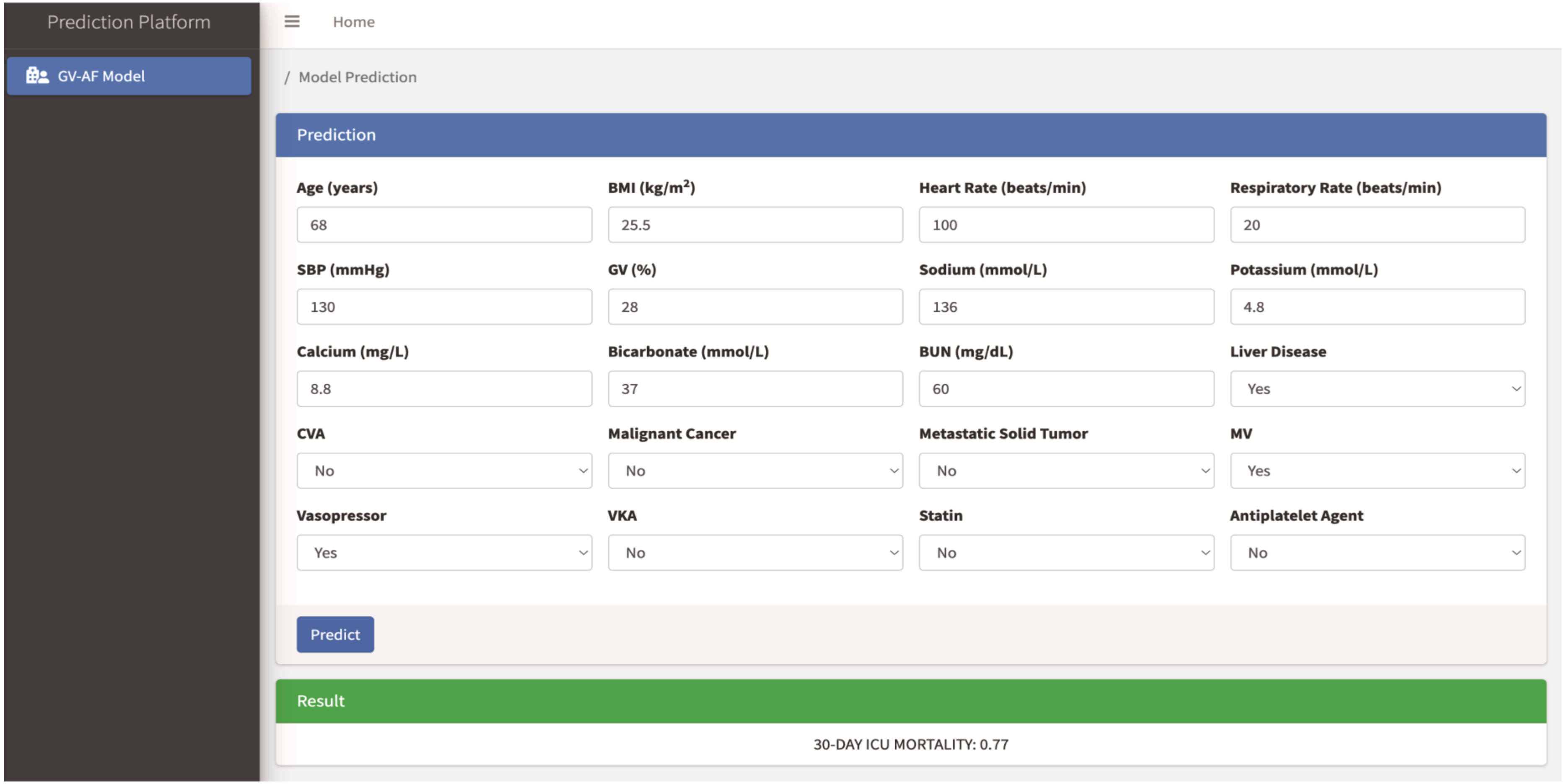

Supplementary Fig. S7 Online platform for the light gradient boosting machine model. BMI, body mass index; BUN, blood urea nitrogen; CVA, cerebrovascular accident; GV, glycaemic variability; MV, mechanical ventilation; SBP, systolic blood pressure; VKA, vitamin K antagonist.
